# Synthesis and crystal structure analysis of (3a*RS*,6*RS*,7a*RS*)-*N*-(4-bromo­phen­yl)-1,6,7,7a-tetra­hydro-3a,6-ep­oxy­iso­indole-2(3*H*)-carboseleno­amide

**DOI:** 10.1107/S2056989026004299

**Published:** 2026-05-07

**Authors:** Dimitrii M. Shchevnikov, Atash V. Gurbanov, Victor N. Khrustalev, Menberu Mengesha Woldemariam, Tuncer Hökelek, Roman A. Litvinov

**Affiliations:** a RUDN University, 6 Miklukho-Maklaya St., Moscow 117198, Russian Federation; b Excellence Center, Baku State University, Z. Khalilov Str. 33, AZ 1148, Baku, Azerbaijan; c Zelinsky Institute of Organic Chemistry of RAS, Leninsky Prospect 47, Moscow 119991, Russian Federation; d Department of Physics, Jimma University, Jimma, Ethiopia; e Hacettepe University, Department of Physics, 06800 Beytepe-Ankara, Türkiye; f Volgograd State Medical University, 1, Pl. Pavshikh Bortsov Square, Volgograd 400131, Russian Federation; g LLC "InnoVVita", Office 401, Room 2, 6 Komsomolskaya St., Volgograd 400066, Russian Federation; Illinois State University, USA

**Keywords:** iso­indole, crystal structure, non-covalent inter­actions

## Abstract

The asymmetric unit of the title compound, C_15_H_15_BrN_2_OSe, contains two crystallographically independent mol­ecules in which the cyclo­hexene and pyrrole rings are in boat and envelope conformations, respectively. In the crystal, C—H⋯O and N–H⋯Se hydrogen bonds link the mol­ecules into [100] chains, enclosing *R*^2^_2_(20), *R*^3^_3_(18) and *R*^4^_4_(4) ring motifs. C—H⋯π(ring) inter­actions help to consolidate the packing. Hirshfeld surface analysis revealed that the most important contributions to the crystal packing are from H⋯H, H⋯C/C⋯H, H⋯Br/Br⋯H and H⋯Se/Se⋯H inter­actions.

## Abstract 2

There are two crystallographically independent mol­ecules in the asymmetric unit of the title compound in which the cyclo­hexene and pyrrole rings are in boat and envelope conformations, respectively. In the crystal, C—H⋯O and N—H⋯Se hydrogen bonds link the mol­ecules into [100] chains. C—H⋯π(ring) inter­actions help to consolidate the packing.

## 1. Chemical context

Fibrotic diseases contribute to global mortality (Mutsaers *et al.*, 2023 ▸) and are poorly reversible (Wang *et al.*, 2024 ▸). Oxidative stress is a recognized driver of fibrosis progression (Cheresh *et al.*, 2013 ▸). Scavenging reactive oxygen species (ROS) with anti­oxidants can reduce fibrosis (Morry *et al.*, 2017 ▸). Iso­indole-based scaffolds are of inter­est as platforms for the development of new anti­oxidant therapeutics, given evidence of anti­oxidant activity in certain members of this class (Yakan *et al.*, 2023 ▸). Epoxidation of the iso­indole core represents a promising avenue for mol­ecular design. Accordingly, the anti­oxidant properties of such compounds are of inter­est. Early studies have shown that hydrogenated iso­indole-7-carb­oxy­lic acids can inhibit protein glycation (Ibragimova *et al.*, 2024 ▸), a process mechanistically linked to oxidative stress (Cho *et al.*, 2007 ▸). Recent studies have shown that the introduction of an N-substituted iso­indole moiety to a chromone scaffold could produce polycyclic compounds possessing significant anti­bacterial properties, especially against Gram-negative bacteria, such as *E. coli* (Parida *et al.*, 2025 ▸). Organoselenium compounds have long been studied for their biological activities, having been shown to exhibit anti­oxidant (Batabyal *et al.*, 2024 ▸) and anti­cancer (Ahn *et al.*, 2006 ▸) activities, as well as acting as insulin analogues, cytostatic agents and uridine phospho­lipase inhibitors.

**Figure 1:**
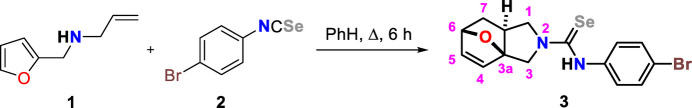
Reaction scheme for the title compound (**3**).

One of the fields of synthetic organic chemistry currently attracting the most attention is the search for synergy of bioactivity in poly-pharmacophoric compounds. Such inter­est drove us to seek ways of combining seleno­urea and iso­indole moieties into a single entity. Building on the iso­indole core, subsequent elaboration led to the development of (3a*RS*,6*RS*,7a*RS*)-*N*-(4-bromo­phen­yl)-1,6,7,7a-tetra­hydro-3a,6-ep­oxy­iso­indole-2(3*H*)-carbo­seleno­amide (**3**), a new and promising representative of the series. The attached selenium atom can participate in inter­molecular chalcogen bonding in the crystal packing of **3** (Gurbanov *et al.*, 2020 ▸, 2022 ▸, 2023 ▸). The seleno­derivative (**3**) was prepared in one stage from commercially available allyl­furfuryl­amine (**1**) and 1-bromo-4-iso­seleno­cyanato­benzene (**2**) (Fig. 1 ▸). The inter­mediate open-chain carbo­seleno­amide underwent fast thermic inter­molecular [4 + 2] cyclo­addition of the allyl moiety to the furan fragment (the IMDAF reaction) to give the cyclic product (**3**) (Nadirova *et al.*, 2021 ▸; Zubkov *et al.*, 2009 ▸). The structure of the target mol­ecule was additionally confirmed using NMR, including spectra on ^77^Se nuclei. All NMR spectra of (**3**) are complicated by amide tautomerism, which occurs in the mol­ecule due to the difficult rotations of fragments around N—C(Se) bonds. Herein, we report the synthesis and mol­ecular and crystal structures of compound (**3**) together with a Hirshfeld surface analysis.
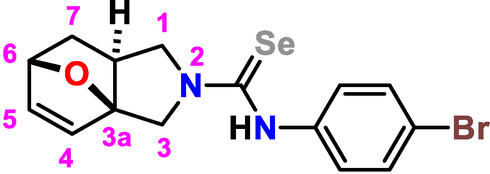


## 2. Structural commentary

**Figure 2:**
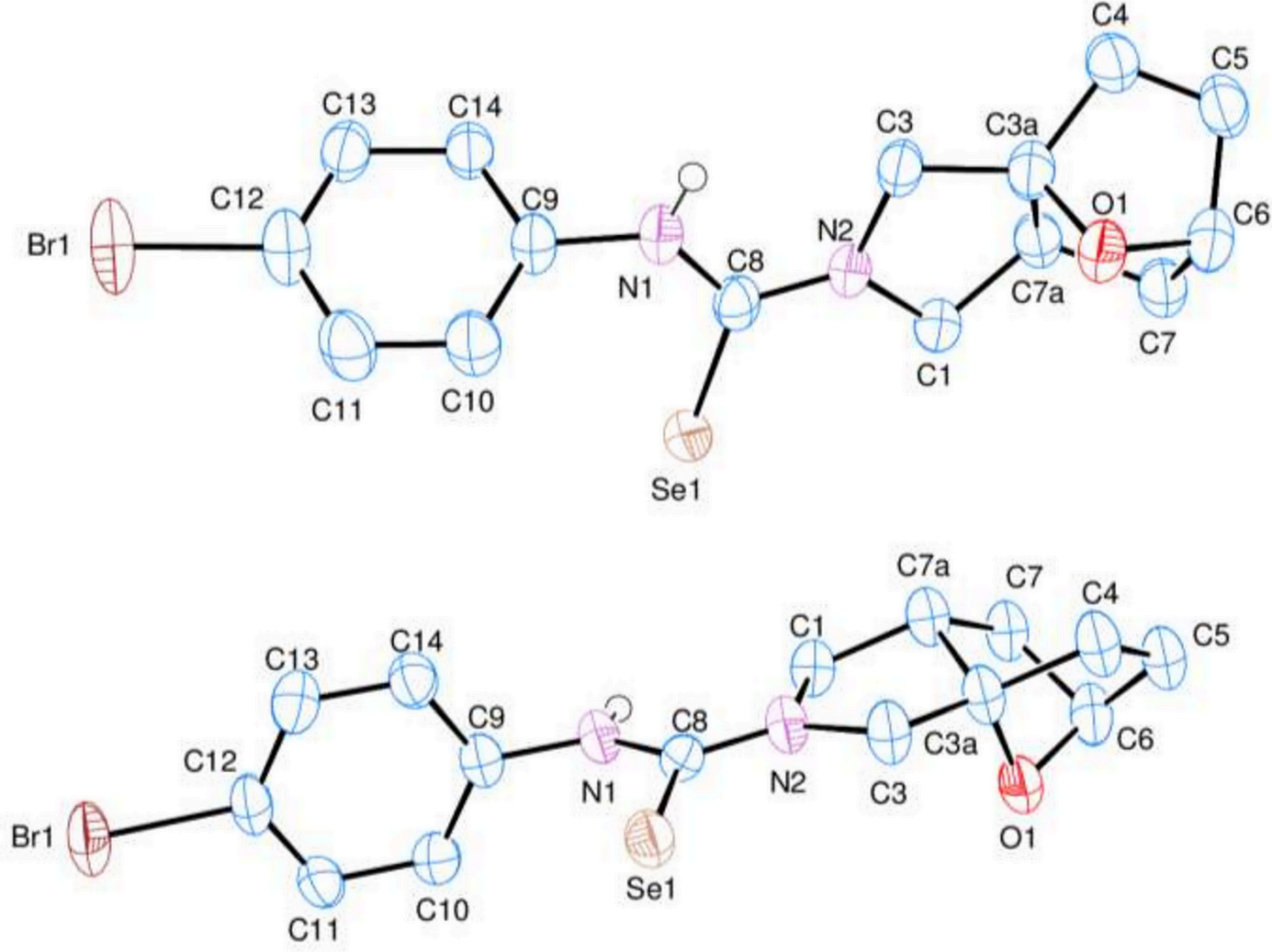
The asymmetric unit of the title compound (**3**) with atom-numbering scheme and 50% probability ellipsoids.

**Figure 3:**
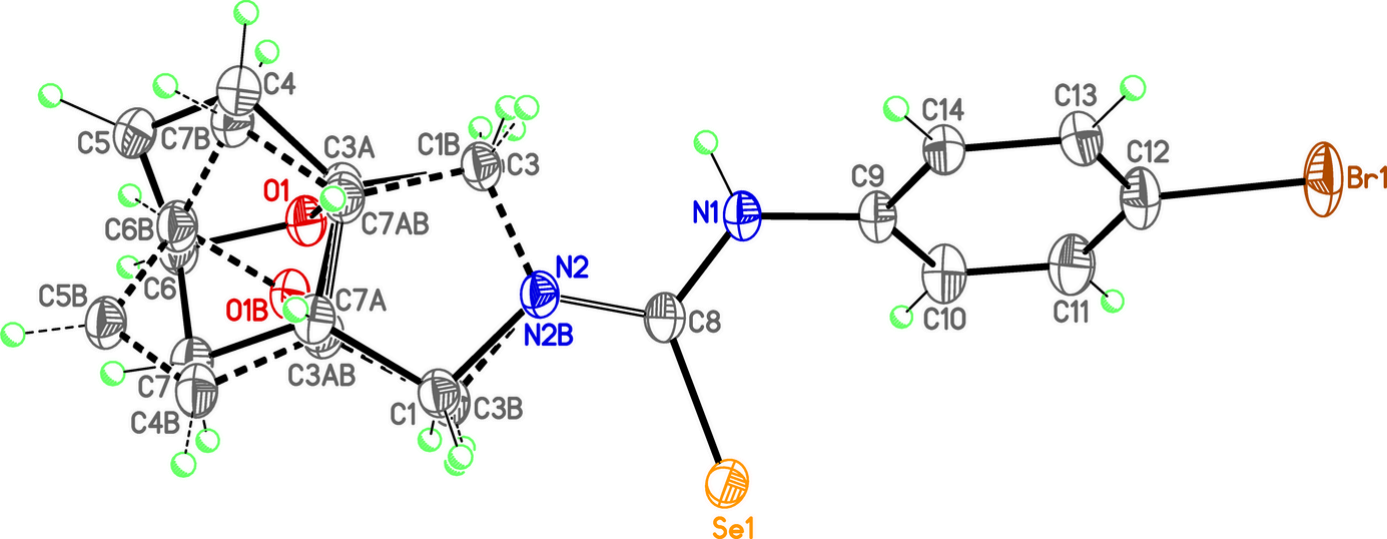
The mol­ecular diagram drawn only for mol­ecule **I** in the asymmetric unit showing the disordering in the ep­oxy­iso­indole fragment over two sets of sites.

**Figure 4:**
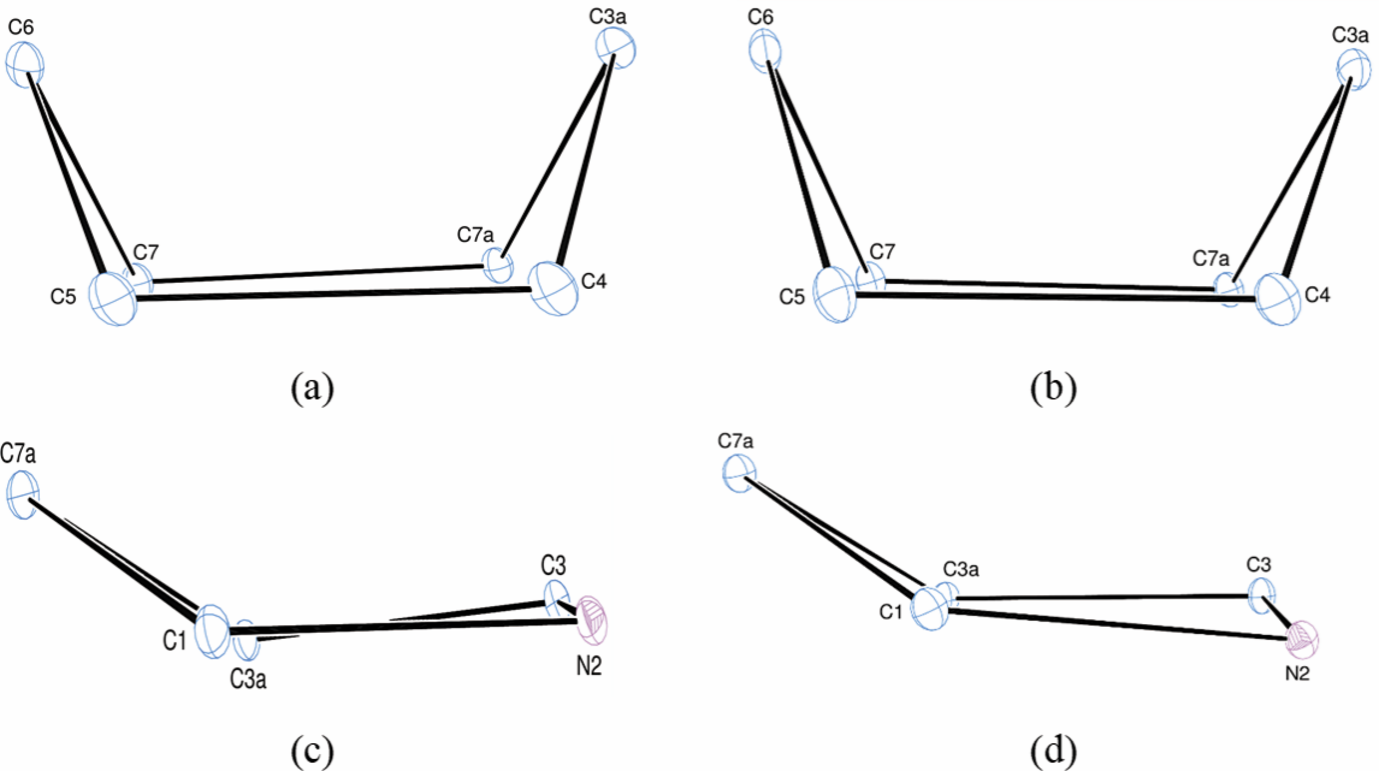
Conformations of the (*a*) cyclo­hexene (in **I**), (*b*) cyclo­hexene (in **II**), (*c*) pyrrole (in **I**) and (*d*) pyrrole (in **II**) rings.

The two independent molecules in the asymmetric unit of the title compound (**3**) contains two crystallographically independent mol­ecules (Fig. 2 ▸), in which the two ep­oxy­iso­indole fragments are disordered over two sets of sites (Fig. 3 ▸). In mol­ecules **I** and **II**, the planar phenyl (C9–C14) rings are oriented at a dihedral angle of 47.84 (5)°. The Br1 atoms are −0.0688 (6) Å (in **I**) and 0.0335 (5) Å (in **II**) away from the corresponding ring planes. The six-membered non-planar (C3*A*/C4–C7/C7*A*) rings are in boat conformations with puckering parameters *Q*_T_ = 0.944 (9) Å, θ = 90.8 (5)° and φ = 359.4 (6)° for mol­ecule **I** and *Q*_T_ = 0.941 (8) Å, θ = 90.3 (5)° and φ = 1.0 (5)° for mol­ecule **II** (Fig. 4 ▸*a* and *b*). On the other hand, the five-membered non-planar (C1/C3/C3*A*/C7*A*/N2) (Fig. 4 ▸*c* and *d*), (O1/C3*A*/C4–C6) and (O1/C3*A*/C6/C7/C7*A*) rings are in envelope conformations with puckering parameters φ = 82.4 (18)° in **I** and 88.6 (15)° in **II**, φ = 2.2 (11)° in **I** and 359.4 (9)° in **II** and φ = 180.4 (9)° in **I** and 181.5 (8)° in **II**, where atoms C7*A*, O1 and O1 are at the flap positions and are −0.5008 (5), 0.7747 (6) and −0.8521 (6) Å, respectively, in **I** and −0.4787 (5), −0.7659 (6) and −0.8543 (6) Å, respectively, in **II** away from the best least-squares planes of the other four atoms of the corresponding rings. There are no significant differences between bond lengths in mol­ecules **I** and **II** but some angles differ significantly, *viz*. C8—N1—C9 [126.4 (5) and 123.3 (4)°], C8—N2—C1 [124.7 (6) and 123.8 (5)°], C3—N2—C1 [111.4 (5) and 112.4 (5)°], N2—C8—N1 [117.0 (5) and 118.0 (5)°], N1—C8—Se1 [121.9 (4) and 120.5 (4)°], C14—C9—C10 [119.8 (5) and 121.3 (5)°], C14—C9—N1 [118.6 (5) and 119.3 (5)°], C10—C9—N1 [121.5 (5) and 119.4 (5)°], C11—C10—C9 [120.0 (5) and 119.3 (5)°] and C13—C12—C11 [121.8 (5) and 122.0 (5)°].

**Figure 5:**
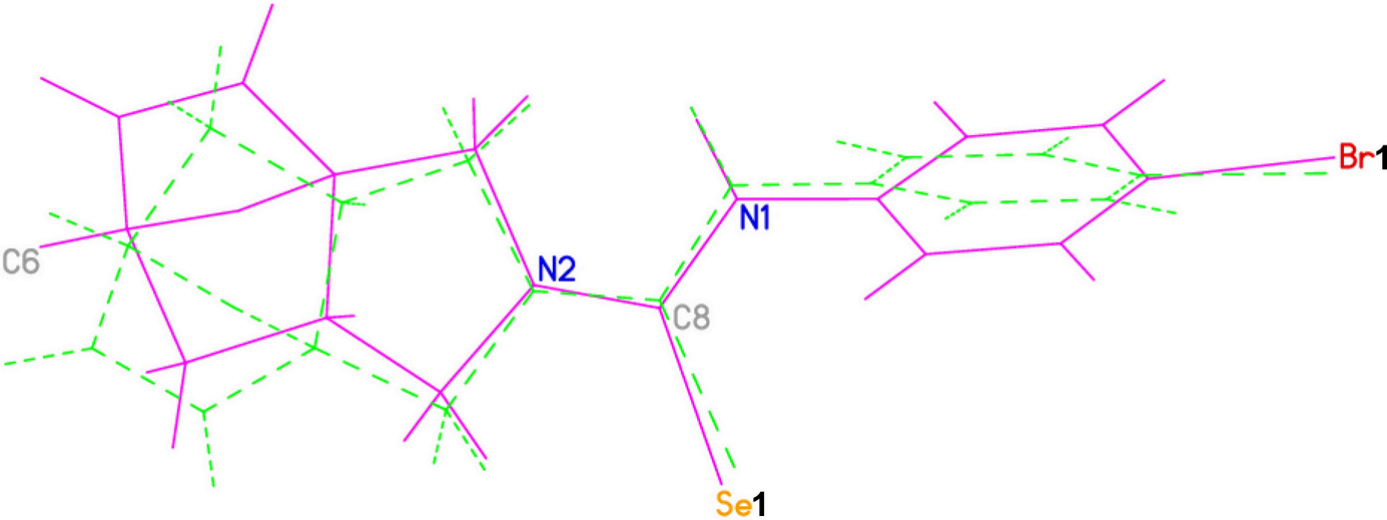
An overlay plot of the two mol­ecules (**I** and **II**) present in the asymmetric unit.

Both ep­oxy­iso­indole fragments are disordered over two sets of sites. Atoms C1, N2, C3, C3*A*, C4, C5, C6, C7, C7*A*, O1, H1*A*, H1*B*, H3*A*, H3*B*, H4, H5, H6, H7*A*, H7*B* and H7*AA* are disordered over two positions in both mol­ecules **I** and **II** and they were refined with occupancy ratios of 0.725 (7):0.275 (7) and 0.831 (6):0.169 (6), respectively. Refinement of this disorder resulted in a meaningful model lowering the previous large difference electron density from 1.685 e.Å^−3^ to 1.381 e.Å^−3^. On the other hand, the large residuals are now limited to the area around Se atoms, and the *R* value converged to 0.0699 instead of 0.0741. For a clearer comparison of the two mol­ecules present in the asymmetric unit, an overlay plot is given in Fig. 5 ▸. The differences between the two mol­ecules are clearly seen in the conformations about the carbo­seleno­amide moieties, torsion angles C9—N1—C8—N2 [171.6 (5) and 175.2 (5)°], C3—N2—C8—N1 [3.4 (9) and 174.4 (6)°], C1—N2—C8—N1 [−173.5 (6) and 0.1 (10)°], C10—C9—N1—C8 [67.9 (8) and −98.1 (6)°] and C14—C9—N1—C8 [−115.1 (6) and 83.8 (7)°] for mol­ecules **I** and **II**, respectively, so that none of the rings overlap exactly.

**Figure 6:**
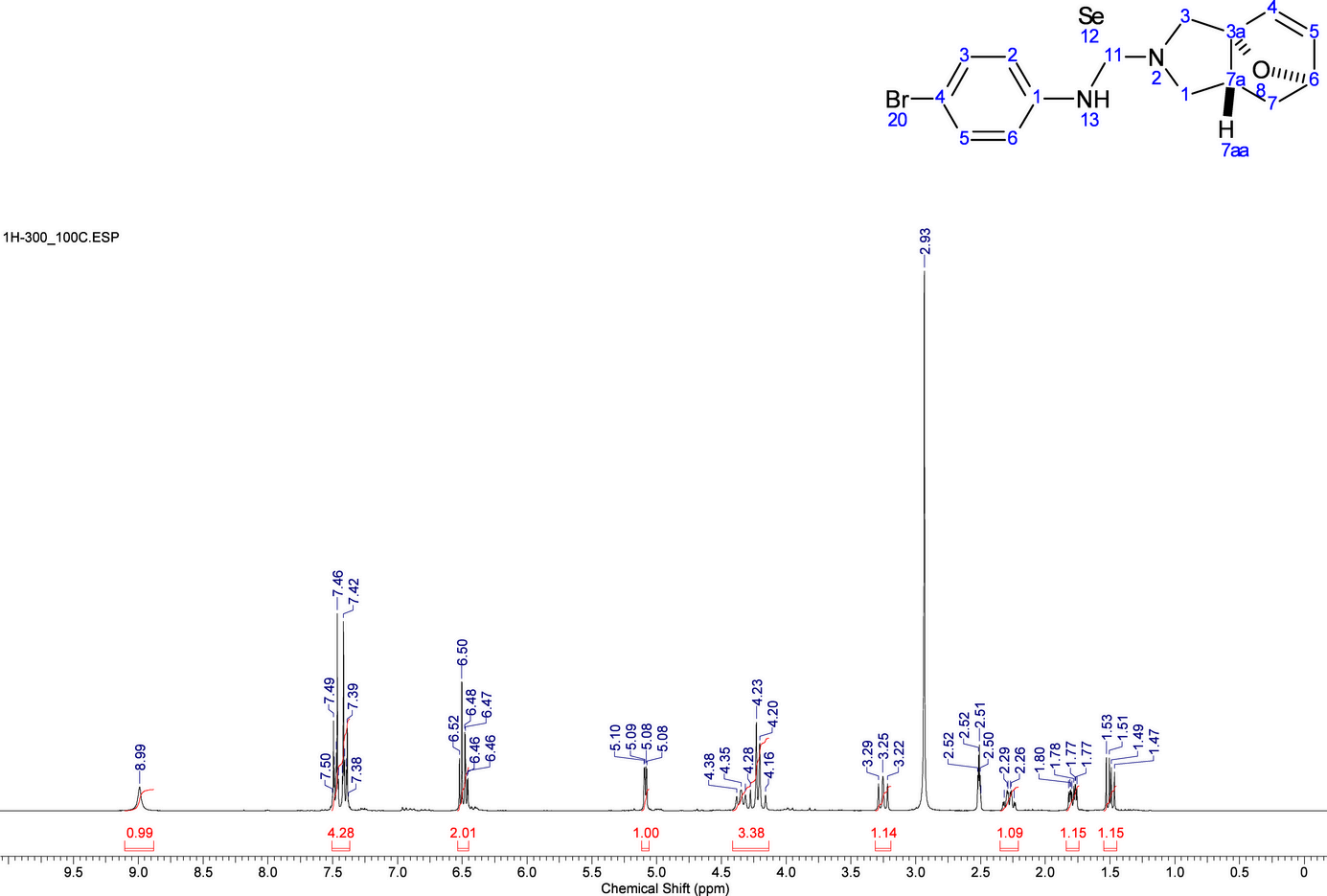
The NMR spectrum recorded at elevated temperatures due to the poor solubility of the title compound (**3**).

Due to the poor solubility of the title compound (**3**), elevated temperatures were required to record the NMR spectra. The sample was heated to ensure complete dissolution (Fig. 6 ▸).

## 3. Supra­molecular features

**Figure 7:**
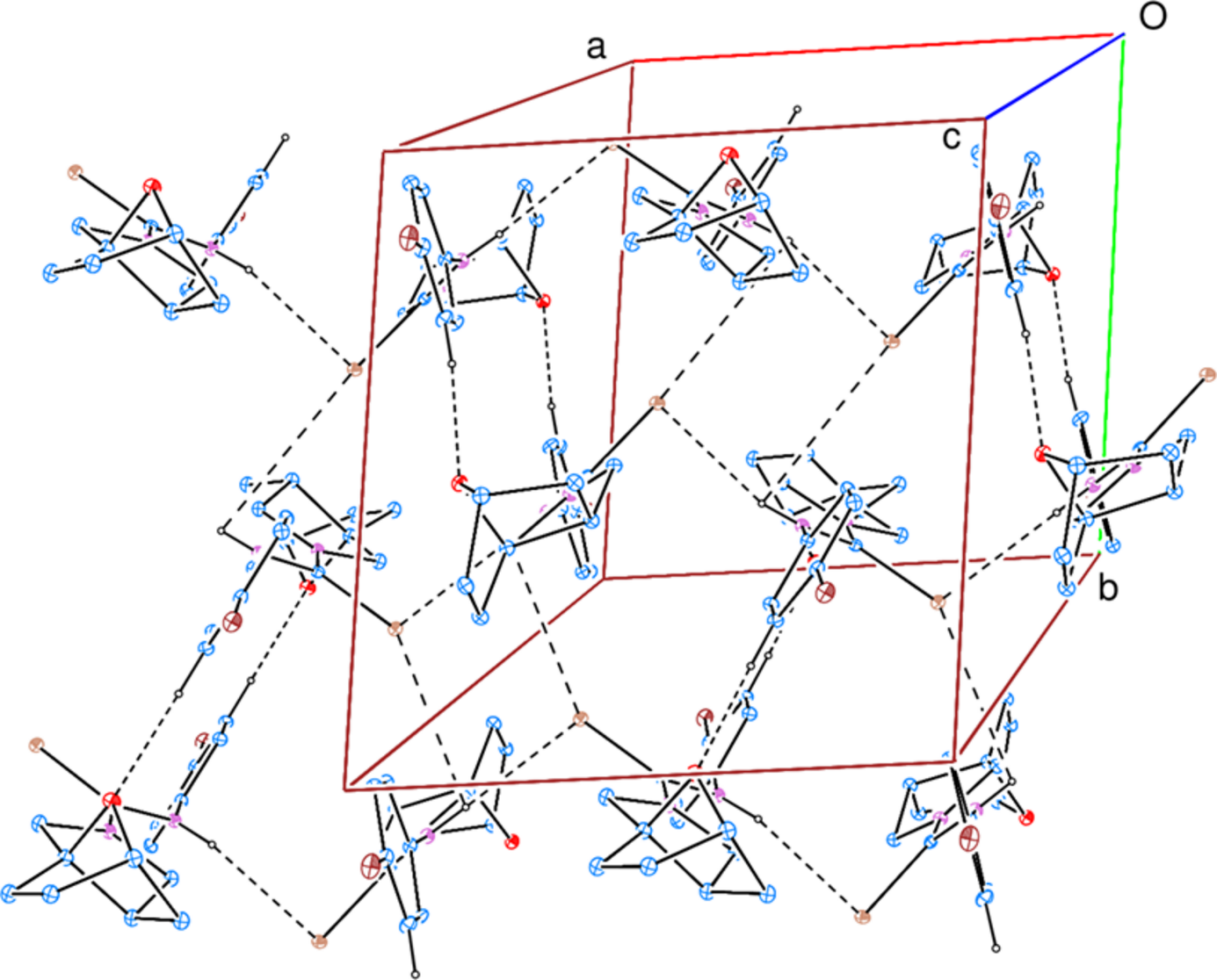
A partial packing diagram of the title compound (**3**). Inter­molecular C—H⋯O and N—H⋯Se hydrogen bonds are shown as dashed lines. H atoms not involved in these inter­actions have been omitted for clarity.

**Figure 8:**
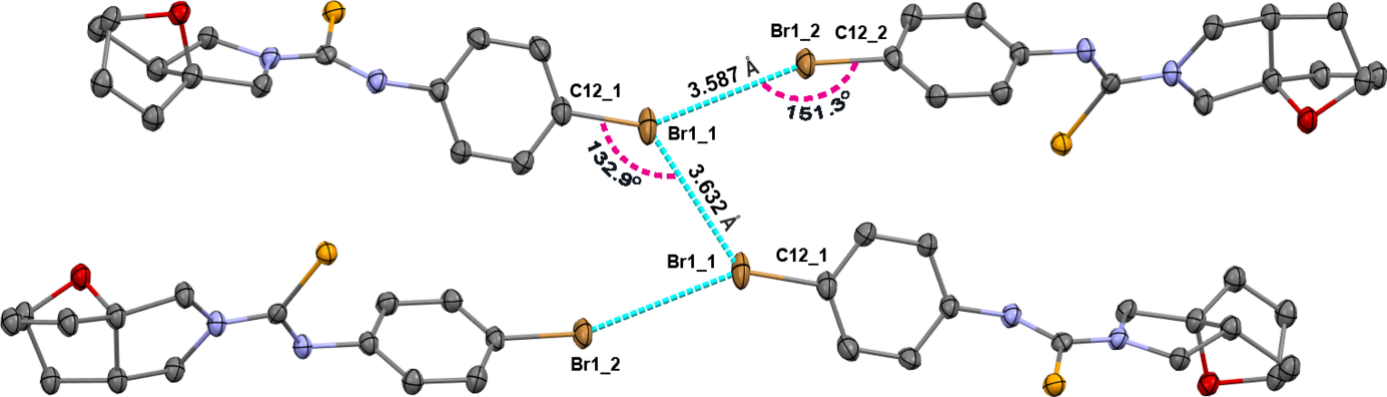
The inter­molecular Br⋯Br halogen bonds leading to a supra­molecular tetra­mer.

In the crystal, inter­molecular C—H⋯O and N—H⋯Se hydrogen bonds (Table 1 ▸) link the mol­ecules into [100] chains, enclosing 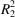
(20), 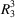
(18) and 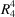
(4) ring motifs (Etter *et al.*, 1990 ▸) (Fig. 7 ▸). C—H⋯π(ring) inter­actions (Table 1 ▸) help to consolidate the packing. Br⋯Br halogen bonds [3.5873 (1) and 3.6318 (12) Å] that are slightly lower than the sum of van der Waals radii of the Br atoms (3.70 Å) occur, leading to a supra­molecular tetra­mer (Fig. 8 ▸). Because of the weak nature of the Br⋯Br inter­actions, the C—Br⋯Br angles [132.9 (2) and 151.3 (2)°] are far from 180°, the directionality term of halogen bonding.

## 4. Hirshfeld surface analysis

**Figure 9:**
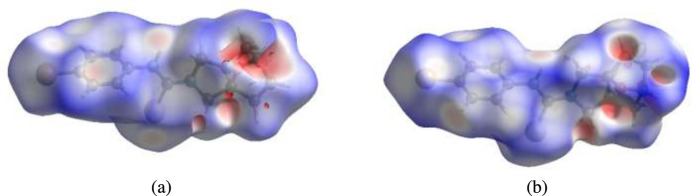
Views of the three-dimensional Hirshfeld surfaces for mol­ecules (*a*) **I** and (*b*) **II** plotted over *d*_norm_.

**Figure 10:**
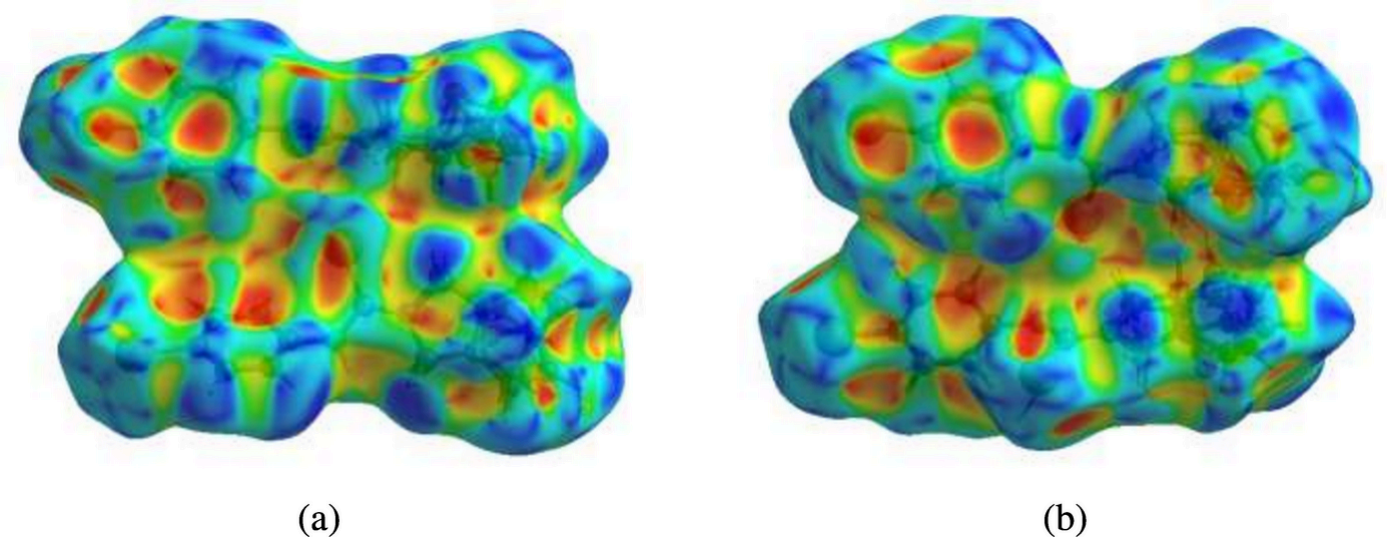
Hirshfeld surfaces for mol­ecules **I** and **II** plotted over shape-index for two orientations showing the C—H⋯π(ring) inter­actions.

**Figure 11:**
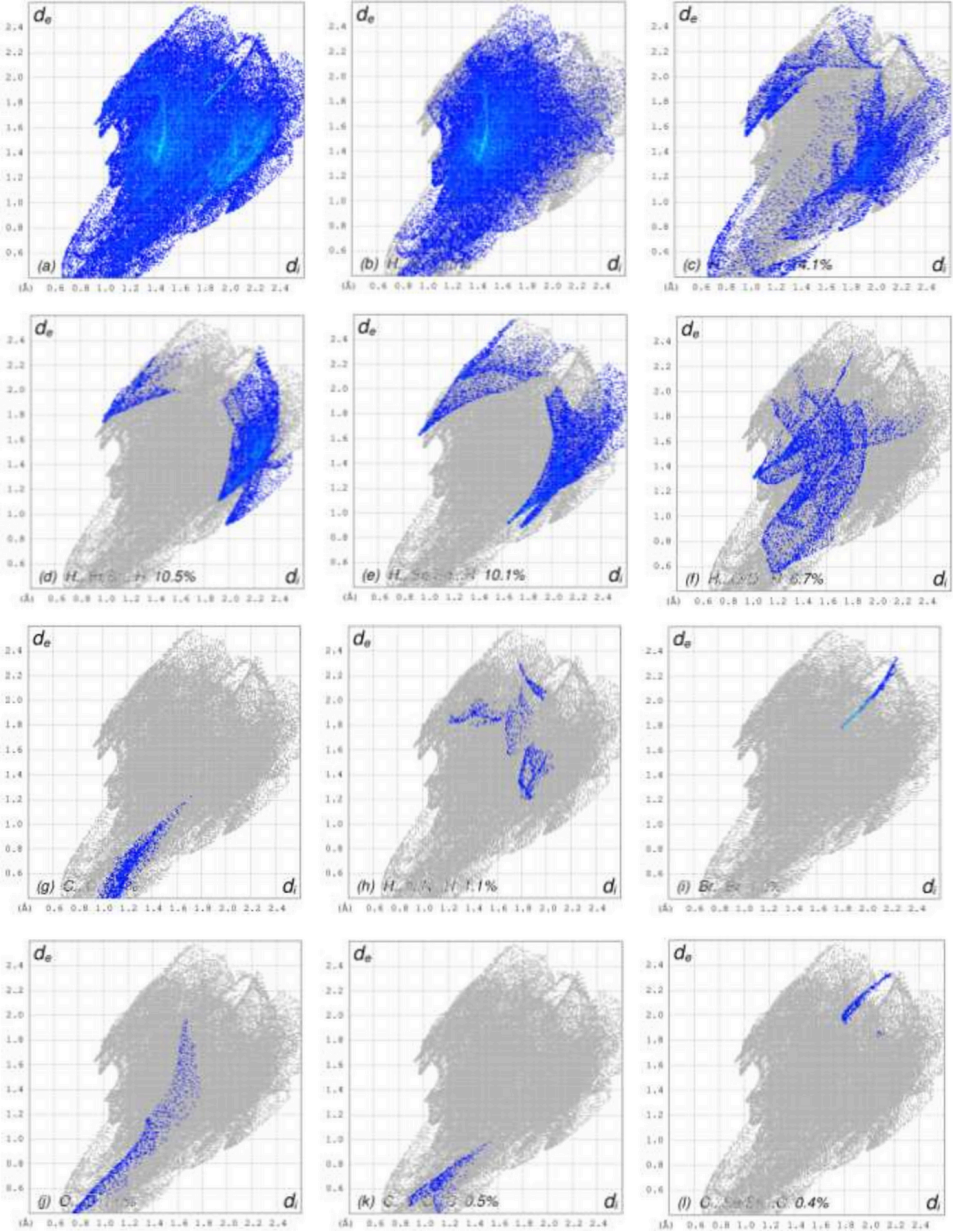
The full two-dimensional fingerprint plots for mol­ecule **I**, showing (*a*) all inter­actions, and delineated into (*b*) H⋯H, (*c*) H⋯C/C⋯H, (*d*) H⋯Br/Br⋯H, (*e*) H⋯Se/Se⋯H, (*f*) H⋯O/O⋯H, (*g*) C⋯C, (*h*) H⋯N/N⋯H, (i) Br⋯Br, (*j*) O⋯O, (*k*) C⋯O/O⋯C, (*l*) C⋯Se/Se⋯C, (*m*) N⋯Se/Se⋯N, (*n*) Se⋯Se and (*o*) O⋯Br/Br⋯O inter­actions. The *d*_i_ and *d*_e_ values are the closest inter­nal and external distances (in Å) from given points on the Hirshfeld surface contacts.

To visualize the inter­molecular inter­actions in the crystal of the title compound, a Hirshfeld surface (HS) analysis was carried out using *Crystal Explorer 17.5* (Spackman *et al.*, 2021 ▸). It is noted that only the major components of the disordered parts of the ep­oxy­iso­indole fragments were taken into account for the analysis. In the HS plotted over *d*_norm_ (Fig. 9 ▸*a* and *b*), the contact distances equal, shorter and longer with respect to the sum of van der Waals radii are shown by the white, red and blue colours, respectively. The red spots indicate their roles as the respective donors and/or acceptors in hydrogen bonding, as discussed. In addition, the shape-index surface was used to identify possible π–π stacking and C—H⋯π(ring) inter­actions as ‘red π-holes’, which are related to the electron ring inter­actions between the C—H groups with the centroid of the aromatic rings of the neighboring mol­ecules. Fig. 10 ▸ clearly suggests that there are C—H⋯π(ring) inter­actions in the title compound but no π–π inter­actions. The overall two-dimensional fingerprint plots are shown in Fig. 11 ▸*a* and 12 ▸*a* and those delineated into H⋯H, H⋯C/C⋯H, H⋯Br/Br⋯H, H⋯Se/Se⋯H, H⋯O/O⋯H, C⋯C, H⋯N/N⋯H, Br⋯Br, O⋯O, C⋯O/O⋯C, C⋯Se/Se⋯C, N⋯Se/Se⋯N, Se⋯Se and O⋯Br/Br⋯O inter­actions (for mol­ecule **I**) and H⋯H, H⋯C/C⋯H, H⋯Se/Se⋯H, H⋯Br/Br⋯H, H⋯O/O⋯H, C⋯C, Br⋯Br, H⋯N/N⋯H, O⋯O, C⋯O/O⋯C, N⋯Se/Se⋯N and C⋯Se/Se⋯C (for mol­ecule **II**) inter­actions are illustrated in Fig. 11 ▸(*b*)–(*l*) and 12(*b*)–(*m*) for mol­ecules **1** and **2**, respectively. Their contributions to the HS are presented in Table 2 ▸. Comparison of the percentage contributions for mol­ecules **I** and **II** shows that there are no significant differences.

## 5. Synthesis and crystallization

*N*-(Furan-2-ylmeth­yl)prop-2-en-1-amine (**1**) (100 mg, 0.7 mmol) was dissolved in benzene (5 ml) at r.t. 1-Bromo-4-iso­seleno­cyanato­benzene (**2**) (190 mg, 0.7 mmol) was added to the solution and the reaction was refluxed for 6 h (TLC control). The resulting mixture was cooled, and the formation of a solid was observed. The crystals were filtered off, washed with diethyl ether (3 × 5 ml), dried under vacuum and then in air. The target product (**3**) did not require further purification; yield 44%, 122.9 mg (0.321 mmol), colourless crystals, m.p. 490–491 K. Single crystals of the title compound were grown from a mixture of EtOH/DMF. IR (KBr), ν (cm^−1^): 3142, 1530, ^1^H NMR (300.1 MHz, DMSO-*d*_6_, 373 K) (*J*, Hz): δ 8.99 (*br.s*, 1H), 7.50–7.38 (*m*, 4H), 6.51 (*d*, *J* = 5.7 Hz, 1H), 6.46 (*dd*, *J* = 5.7, 1.7 Hz, 1H), 5.08 (*dd*, *J* = 4.4, 1.7 Hz, 1H), 4.38–4.16 (*m*, 3H), 3.25 (*br.dd*, *J* = 11.4, 9.7 Hz, 1H), 2.33–2.23 (*m*, 1H), 1.79 (*ddd*, *J* = 11.7, 4.4, 2.8 Hz, 1H), 1.50 (*dd*, *J* = 11.7, 7.5 Hz, 1H) ppm. ^13^C{^1^H} NMR (75.5 MHz, DMSO-*d*_6_, 373 K): δ 178.2, 141.3, 137.8, 134.3, 131.1 (2C), 128.9 (2C), 122.0, 94.1, 80.2, 57.3, 54.0, 41.5, 32.3. ^77^Se{^1^H} NMR (57.2 MHz, DMSO-*d*_6_, 373 K): δ 303.0. ^1^H NMR (700.2 MHz, DMSO-*d*_6_, 353 K): (*J*, Hz): δ 9.12 (*br.s*, 1H), 7.49 (*d*, *J* = 8.1 Hz, 2H), 7.38 (*d*, *J* = 8.1 Hz, 2H), 6.52 (*br.d*, *J* = 5.2 Hz, 1H), 6.48 (*br.d*, *J* = 5.2 Hz, 1H), 5.06 (*br.d*, *J* = 2.9 Hz, 1H), broaden H-1,3 signals ∼4.50–4.00 (*m*, 3H), 3.23 (*br.s*, 1H), 2.28 (*br.s*, 1H), 1.78 (*br.d*, *J* = 10.5 Hz, 1H), 1.49 (*br.d*, *J* = 11.0, 7.6 Hz, 1H) ppm. ^13^C NMR (176.1 MHz, DMSO-*d*_6_, 353 K): δ signals of 4 carbon atoms of the ep­oxy­iso­indole moiety are very broad and absent in the spectra, 177.8, 141.3, 137.8, 134.3, 131.2 (2C), 129.0 (2C), 117.9 80.2, 32.3. ^77^Se{^1^H} NMR (57.2 MHz, DMSO-d_6_): δ the signal of the Se nuclei are duplicated due to amide rotamerism 286.0, 283.5 ppm. MS (ESI) *m*/*z*: [*M*]^+^ 399 [*M* + H]^+^.

## 6. Refinement

Crystal data, data collection and structure refinement details are summarized in Table 3 ▸. The N- and C-bound hydrogen-atom positions were calculated geometrically at distances of 0.88 (for NH), 1.00 (for methine CH), 0.95 (for aromatic CH) and 0.99 Å (for methyl­ene CH) and refined using a riding model by applying the constraint *U*_iso_(H) = 1.2*U*_eq_(C,N).

## Supplementary Material

Crystal structure: contains datablock(s) I, global. DOI: 10.1107/S2056989026004299/ej2017sup1.cif

Structure factors: contains datablock(s) I. DOI: 10.1107/S2056989026004299/ej2017Isup5.hkl

checkcif. DOI: 10.1107/S2056989026004299/ej2017sup3.pdf

Supporting information file. DOI: 10.1107/S2056989026004299/ej2017sup4.pdf

Supporting information file. DOI: 10.1107/S2056989026004299/ej2017Isup5.cml

CCDC reference: 2549118

Additional supporting information:  crystallographic information; 3D view; checkCIF report

## Figures and Tables

**Figure 12:**
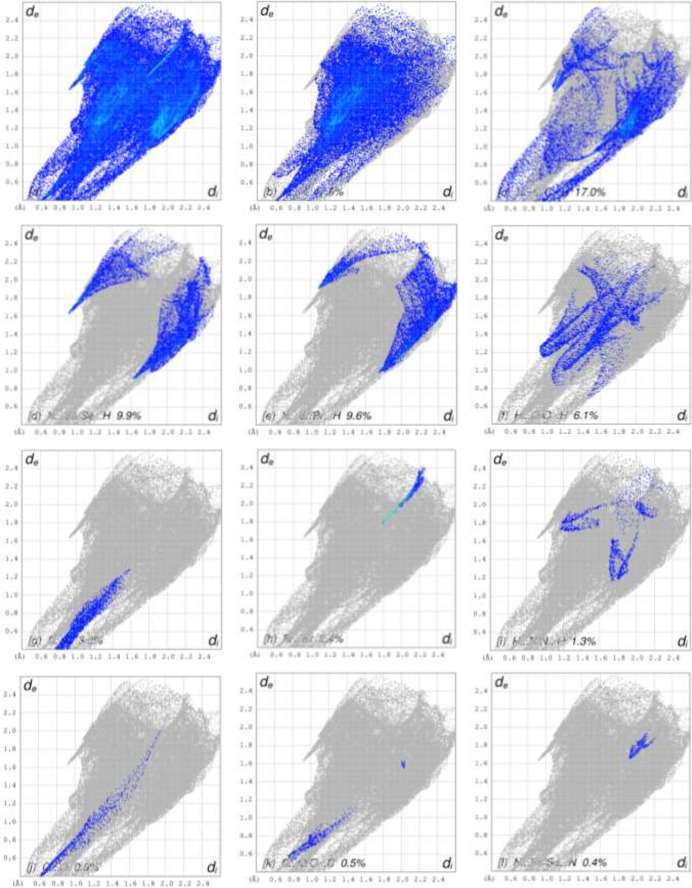
The full two-dimensional fingerprint plots for mol­ecule **II**, showing (*a*) all inter­actions, and delineated into (*b*) H⋯H, (*c*) H⋯C/C⋯H, (*d*) H⋯Se/Se⋯H, (*e*) H⋯Br/Br⋯H, (*f*) H⋯O/O⋯H, (*g*) C⋯C, (*h*) Br⋯Br, (i) H⋯N/N⋯H, (*j*) O⋯O, (*k*) C⋯O/O⋯C, (*l*) N⋯Se/Se⋯N and (*m*) C⋯Se/Se⋯C inter­actions. The *d*_i_ and *d*_e_ values are the closest inter­nal and external distances (in Å) from given points on the Hirshfeld surface.

**Table 1:** Hydrogen-bond geometry (Å, °) *Cg*8 and *Cg*17 are the centroids of the C9_1–C14_1 and C9_2–C14_2 rings, respectively.

*D*—H⋯*A*	*D*—H	H⋯*A*	*D*⋯*A*	*D*—H⋯*A*
N1_1—H1_1⋯Se1_2^i^	0.88	2.67	3.460 (5)	151
C11_1—H11_1⋯O1_1^i^	0.95	2.34	3.283 (8)	174
C11_1—H11_1⋯O1*B*_1^i^	0.95	2.25	3.176 (14)	165
N1_2—H1_2⋯Se1_1^ii^	0.88	2.64	3.393 (5)	144
C11_2—H11_2⋯O1_2^iii^	0.95	2.44	3.368 (7)	167
C11_2—H11_2⋯O1*B*_2^iii^	0.95	2.48	3.36 (2)	154
C1_1—H1*A*_1⋯*Cg*17^ii^	0.99	2.83	3.720 (11)	150
C3_2—H3*B*_2⋯*Cg*8^i^	0.99	2.81	3.724 (9)	154

Symmetry codes: (i) 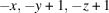; (ii) 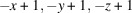; (iii) 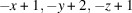.

**Table 2:** Comparison of the percentage contributions for various inter­actions in mol­ecules **1** and **2**

Contacts	**1**	**2**
H⋯H	51.5	47.6
H⋯C/C⋯H	14.1	17.0
H⋯Br/Br⋯H	10.5	9.6
H⋯Se/Se⋯H	10.1	9.9
H⋯O/O⋯H	6.7	6.1
C⋯C	2.4	3.8
H⋯N/N⋯H	1.1	1.3
Br.·Br	1.0	2.4
O⋯O	1.0	0.9
C⋯O/O⋯C	0.5	0.5
C⋯Se/Se⋯C	0.4	0.4
N⋯Se/Se⋯N	0.3	0.4
Se⋯Se	0.2	0.0
O⋯Br/Br⋯O	0.1	0.0

**Table 3:** Experimental details

Crystal data	
Chemical formula	C_15_H_15_BrN_2_OSe
*M*_r_	398.15
Crystal system, space group	Triclinic, *P* 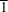
Temperature (K)	100
*a*, *b*, *c* (Å)	9.7367 (4), 10.3981 (4), 15.7685 (5)
α, β, γ (°)	73.059 (3), 76.870 (3), 84.140 (4)
*V* (Å^3^)	1486.15 (10)
*Z*	4
Radiation type	Cu *K*α
μ (mm^−1^)	6.54
Crystal size (mm)	0.30 × 0.06 × 0.03

Data collection	
Diffractometer	Rigaku XtaLAB Synergy-S, HyPix-6000HE area-detector
Absorption correction	Gaussian (*CrysAlis PRO* (Rigaku OD, 2021 ▸)
*T*_min_, *T*_max_	0.353, 1.000
No. of measured, independent and observed [*I* > 2σ(*I*)] reflections	28318, 6210, 5303
*R*_int_	0.101
(sin θ/λ)_max_ (Å^−1^)	0.639

Refinement	
*R*[*F*^2^ > 2σ(*F*^2^)], *wR*(*F*^2^), *S*	0.070, 0.178, 1.07
No. of reflections	6210
No. of parameters	525
No. of restraints	930
H-atom treatment	H-atom parameters constrained
Δρ_max_, Δρ_min_ (e Å^−3^)	1.38, −1.22

Computer programs: *CrysAlis PRO* (Rigaku OD, 2021 ▸), *SHELXT* (Sheldrick, 2015*a* ▸), *SHELXL* (Sheldrick, 2015*b* ▸) and *SHELXTL* (Sheldrick, 2008 ▸).

## supplementary crystallographic information

### (3aRS,6RS,7aRS)-N-(4-Bromophenyl)-1,6,7,7a-tetrahydro-3a,6-epoxyisoindole-2(3H)-carboselenoamide Crystal data

**Table:**

C_15_H_15_BrN_2_OSe	*Z* = 4
*M**_r_* = 398.15	*F*(000) = 784
Triclinic, *P*1	*D*_x_ = 1.780 Mg m^−^^3^
*a* = 9.7367 (4) Å	Cu *K*α radiation, λ = 1.54184 Å
*b* = 10.3981 (4) Å	Cell parameters from 14508 reflections
*c* = 15.7685 (5) Å	θ = 3.0–79.4°
α = 73.059 (3)°	µ = 6.54 mm^−^^1^
β = 76.870 (3)°	*T* = 100 K
γ = 84.140 (4)°	Prismatic needle, colourless
*V* = 1486.15 (10) Å^3^	0.30 × 0.06 × 0.03 mm

### (3aRS,6RS,7aRS)-N-(4-Bromophenyl)-1,6,7,7a-tetrahydro-3a,6-epoxyisoindole-2(3H)-carboselenoamide Data collection

**Table:**

Rigaku XtaLAB Synergy-S, HyPix-6000HE area-detector diffractometer	5303 reflections with *I* > 2σ(*I*)
Radiation source: micro-focus sealed X-ray tube	*R*_int_ = 0.101
φ and ω scans	θ_max_ = 80.4°, θ_min_ = 3.0°
Absorption correction: gaussian (CrysAlisPro (Rigaku OD, 2021)	*h* = −12→12
*T*_min_ = 0.353, *T*_max_ = 1.000	*k* = −13→12
28318 measured reflections	*l* = −20→20
6210 independent reflections	

### (3aRS,6RS,7aRS)-N-(4-Bromophenyl)-1,6,7,7a-tetrahydro-3a,6-epoxyisoindole-2(3H)-carboselenoamide Refinement

**Table:**

Refinement on *F*^2^	Primary atom site location: difference Fourier map
Least-squares matrix: full	Secondary atom site location: difference Fourier map
*R*[*F*^2^ > 2σ(*F*^2^)] = 0.070	Hydrogen site location: inferred from neighbouring sites
*wR*(*F*^2^) = 0.178	H-atom parameters constrained
*S* = 1.07	*w* = 1/[σ^2^(*F*_o_^2^) + (0.0974*P*)^2^ + 4.6292*P*] where *P* = (*F*_o_^2^ + 2*F*_c_^2^)/3
6210 reflections	(Δ/σ)_max_ = 0.001
525 parameters	Δρ_max_ = 1.38 e Å^−^^3^
930 restraints	Δρ_min_ = −1.22 e Å^−^^3^

### (3aRS,6RS,7aRS)-N-(4-Bromophenyl)-1,6,7,7a-tetrahydro-3a,6-epoxyisoindole-2(3H)-carboselenoamide Special details

**Table:**

Geometry. All esds (except the esd in the dihedral angle between two l.s. planes) are estimated using the full covariance matrix. The cell esds are taken into account individually in the estimation of esds in distances, angles and torsion angles; correlations between esds in cell parameters are only used when they are defined by crystal symmetry. An approximate (isotropic) treatment of cell esds is used for estimating esds involving l.s. planes.

### (3aRS,6RS,7aRS)-N-(4-Bromophenyl)-1,6,7,7a-tetrahydro-3a,6-epoxyisoindole-2(3H)-carboselenoamide Fractional atomic coordinates and isotropic or equivalent isotropic displacement parameters (Å^2^)

**Table:**

	*x*	*y*	*z*	*U*_iso_*/*U*_eq_	Occ. (<1)
Br1_1	−0.00774 (8)	0.15550 (10)	0.91076 (4)	0.0679 (3)	
Se1_1	0.30048 (7)	0.46618 (6)	0.46015 (4)	0.04111 (18)	
N1_1	0.0925 (5)	0.2751 (5)	0.5014 (3)	0.0388 (9)	
H1_1	0.033003	0.235262	0.483781	0.047*	
C1_1	0.3049 (11)	0.4122 (9)	0.2719 (6)	0.0392 (18)	0.725 (7)
H1A_1	0.399434	0.399056	0.287253	0.047*	0.725 (7)
H1B_1	0.282548	0.509933	0.251227	0.047*	0.725 (7)
N2_1	0.1970 (5)	0.3465 (4)	0.3511 (3)	0.0366 (8)	0.725 (7)
C3_1	0.1025 (12)	0.2681 (12)	0.3260 (7)	0.039 (2)	0.725 (7)
H3A_1	0.002571	0.296332	0.344365	0.046*	0.725 (7)
H3B_1	0.114863	0.170718	0.355145	0.046*	0.725 (7)
C3A_1	0.1457 (8)	0.2983 (8)	0.2244 (5)	0.0414 (14)	0.725 (7)
C4_1	0.1306 (11)	0.2055 (9)	0.1700 (6)	0.0455 (17)	0.725 (7)
H4_1	0.116417	0.111466	0.191433	0.055*	0.725 (7)
C5_1	0.1415 (9)	0.2833 (8)	0.0854 (5)	0.0469 (16)	0.725 (7)
H5_1	0.139614	0.255589	0.033326	0.056*	0.725 (7)
C6_1	0.1574 (10)	0.4242 (9)	0.0887 (5)	0.0462 (17)	0.725 (7)
H6_1	0.127456	0.497280	0.038396	0.055*	0.725 (7)
C7_1	0.3096 (9)	0.4342 (9)	0.1010 (5)	0.0459 (15)	0.725 (7)
H7A_1	0.381260	0.398371	0.057215	0.055*	0.725 (7)
H7B_1	0.330864	0.527874	0.095136	0.055*	0.725 (7)
C7A_1	0.3005 (8)	0.3443 (8)	0.1988 (5)	0.0413 (14)	0.725 (7)
H7AA_1	0.369423	0.265808	0.200985	0.050*	0.725 (7)
O1_1	0.0777 (6)	0.4217 (6)	0.1781 (3)	0.0421 (12)	0.725 (7)
C1B_1	0.120 (3)	0.253 (3)	0.3256 (16)	0.039 (3)	0.275 (7)
H1C_1	0.022337	0.287147	0.321875	0.047*	0.275 (7)
H1D_1	0.118223	0.162414	0.368816	0.047*	0.275 (7)
N2B_1	0.1970 (5)	0.3465 (4)	0.3511 (3)	0.0366 (8)	0.275 (7)
C3B_1	0.291 (3)	0.436 (3)	0.2759 (12)	0.040 (3)	0.275 (7)
H3C_1	0.390635	0.419194	0.281893	0.048*	0.275 (7)
H3D_1	0.263991	0.532041	0.271727	0.048*	0.275 (7)
C3AB_1	0.2664 (17)	0.3980 (17)	0.1958 (9)	0.041 (2)	0.275 (7)
C4B_1	0.3772 (18)	0.404 (2)	0.1118 (10)	0.044 (3)	0.275 (7)
H4B_1	0.476242	0.407838	0.105237	0.053*	0.275 (7)
C5B_1	0.3097 (18)	0.401 (2)	0.0490 (10)	0.044 (3)	0.275 (7)
H5B_1	0.349877	0.404278	−0.012295	0.053*	0.275 (7)
C6B_1	0.155 (2)	0.393 (2)	0.0940 (11)	0.044 (2)	0.275 (7)
H6B_1	0.087964	0.426091	0.051986	0.053*	0.275 (7)
C7B_1	0.131 (3)	0.2492 (19)	0.1579 (13)	0.045 (3)	0.275 (7)
H7C_1	0.173925	0.180053	0.126932	0.054*	0.275 (7)
H7D_1	0.029384	0.232814	0.182530	0.054*	0.275 (7)
C7AB_1	0.209 (2)	0.2531 (16)	0.2329 (10)	0.042 (2)	0.275 (7)
H7AB_1	0.287638	0.183335	0.237955	0.051*	0.275 (7)
O1B_1	0.1482 (16)	0.4691 (14)	0.1595 (9)	0.042 (2)	0.275 (7)
C8_1	0.1889 (5)	0.3519 (5)	0.4356 (3)	0.0356 (10)	
C9_1	0.0777 (6)	0.2523 (6)	0.5963 (3)	0.0377 (11)	
C10_1	0.0327 (7)	0.3551 (6)	0.6380 (4)	0.0471 (13)	
H10_1	0.018502	0.444400	0.602203	0.056*	
C11_1	0.0088 (7)	0.3272 (7)	0.7308 (4)	0.0526 (15)	
H11_1	−0.022626	0.396542	0.759604	0.063*	
C12_1	0.0311 (7)	0.1963 (7)	0.7818 (4)	0.0505 (14)	
C13_1	0.0777 (7)	0.0944 (6)	0.7421 (4)	0.0443 (12)	
H13_1	0.092602	0.005481	0.778176	0.053*	
C14_1	0.1025 (6)	0.1231 (6)	0.6486 (4)	0.0389 (11)	
H14_1	0.136740	0.053811	0.620145	0.047*	
Br1_2	0.25531 (8)	0.75885 (8)	0.90577 (4)	0.0552 (2)	
Se1_2	0.18559 (6)	0.92147 (6)	0.47837 (4)	0.03988 (18)	
N1_2	0.4327 (5)	0.7680 (5)	0.5088 (3)	0.0365 (9)	
H1_2	0.515206	0.730673	0.490691	0.044*	
C1_2	0.5323 (10)	0.7390 (13)	0.3349 (5)	0.0397 (13)	0.831 (6)
H1A_2	0.543596	0.652868	0.381026	0.048*	0.831 (6)
H1B_2	0.614456	0.794349	0.325014	0.048*	0.831 (6)
N2_2	0.3984 (5)	0.8117 (5)	0.3640 (3)	0.0376 (9)	0.831 (6)
C3_2	0.3156 (9)	0.8578 (8)	0.2923 (5)	0.0425 (17)	0.831 (6)
H3A_2	0.288142	0.954533	0.282186	0.051*	0.831 (6)
H3B_2	0.229513	0.805507	0.307811	0.051*	0.831 (6)
C3A_2	0.4165 (7)	0.8321 (8)	0.2102 (4)	0.0419 (13)	0.831 (6)
C4_2	0.3733 (8)	0.8112 (8)	0.1289 (4)	0.0475 (15)	0.831 (6)
H4_2	0.283605	0.785900	0.126428	0.057*	0.831 (6)
C5_2	0.4851 (8)	0.8350 (8)	0.0626 (4)	0.0475 (14)	0.831 (6)
H5_2	0.492770	0.831504	0.002160	0.057*	0.831 (6)
C6_2	0.5986 (9)	0.8691 (8)	0.1025 (5)	0.0477 (15)	0.831 (6)
H6_2	0.676085	0.922685	0.057128	0.057*	0.831 (6)
C7_2	0.6492 (8)	0.7391 (8)	0.1676 (4)	0.0450 (14)	0.831 (6)
H7A_2	0.669275	0.663923	0.139352	0.054*	0.831 (6)
H7B_2	0.733960	0.753993	0.187783	0.054*	0.831 (6)
C7A_2	0.5188 (7)	0.7141 (8)	0.2461 (4)	0.0425 (13)	0.831 (6)
H7AA_2	0.478651	0.624617	0.256699	0.051*	0.831 (6)
O1_2	0.5165 (6)	0.9373 (5)	0.1664 (3)	0.0463 (11)	0.831 (6)
C1B_2	0.326 (4)	0.889 (4)	0.2913 (17)	0.039 (3)	0.169 (6)
H1C_2	0.354724	0.984011	0.268051	0.047*	0.169 (6)
H1D_2	0.222209	0.886858	0.311360	0.047*	0.169 (6)
N2B_2	0.3984 (5)	0.8117 (5)	0.3640 (3)	0.0376 (9)	0.169 (6)
C3B_2	0.531 (4)	0.742 (5)	0.3375 (16)	0.039 (3)	0.169 (6)
H3C_2	0.529778	0.644714	0.369489	0.047*	0.169 (6)
H3D_2	0.612221	0.781893	0.347498	0.047*	0.169 (6)
C3AB_2	0.533 (2)	0.768 (2)	0.2383 (12)	0.043 (2)	0.169 (6)
C4B_2	0.598 (3)	0.668 (2)	0.1876 (15)	0.045 (3)	0.169 (6)
H4B_2	0.625006	0.576213	0.211977	0.054*	0.169 (6)
C5B_2	0.610 (3)	0.735 (3)	0.1022 (15)	0.047 (3)	0.169 (6)
H5B_2	0.635581	0.698389	0.051758	0.056*	0.169 (6)
C6B_2	0.575 (3)	0.881 (3)	0.0988 (14)	0.046 (3)	0.169 (6)
H6B_2	0.623663	0.947474	0.043065	0.055*	0.169 (6)
C7B_2	0.412 (3)	0.899 (3)	0.1211 (14)	0.047 (3)	0.169 (6)
H7C_2	0.367144	0.864103	0.082050	0.056*	0.169 (6)
H7D_2	0.380590	0.994072	0.115919	0.056*	0.169 (6)
C7AB_2	0.381 (2)	0.811 (3)	0.2209 (14)	0.044 (3)	0.169 (6)
H7AB_2	0.322772	0.732251	0.230271	0.053*	0.169 (6)
O1B_2	0.604 (2)	0.889 (2)	0.1832 (13)	0.045 (2)	0.169 (6)
C8_2	0.3529 (6)	0.8241 (5)	0.4474 (3)	0.0345 (10)	
C9_2	0.3902 (6)	0.7659 (5)	0.6024 (3)	0.0364 (11)	
C10_2	0.4397 (6)	0.8613 (6)	0.6321 (4)	0.0419 (12)	
H10_2	0.500901	0.927874	0.590903	0.050*	
C11_2	0.3989 (6)	0.8591 (6)	0.7232 (4)	0.0412 (12)	
H11_2	0.430534	0.924927	0.744619	0.049*	
C12_2	0.3117 (6)	0.7595 (6)	0.7819 (3)	0.0396 (12)	
C13_2	0.2630 (6)	0.6619 (7)	0.7527 (4)	0.0460 (13)	
H13_2	0.203282	0.594257	0.794113	0.055*	
C14_2	0.3035 (6)	0.6655 (6)	0.6620 (4)	0.0407 (11)	
H14_2	0.272202	0.599520	0.640586	0.049*	

### (3aRS,6RS,7aRS)-N-(4-Bromophenyl)-1,6,7,7a-tetrahydro-3a,6-epoxyisoindole-2(3H)-carboselenoamide Atomic displacement parameters (Å^2^)

**Table:**

	*U*^11^	*U*^22^	*U*^33^	*U*^12^	*U*^13^	*U*^23^
Br1_1	0.0604 (4)	0.1135 (7)	0.0255 (3)	0.0060 (4)	−0.0040 (3)	−0.0196 (3)
Se1_1	0.0526 (4)	0.0383 (3)	0.0350 (3)	−0.0026 (2)	−0.0133 (2)	−0.0107 (2)
N1_1	0.045 (2)	0.045 (2)	0.026 (2)	−0.0009 (19)	−0.0091 (18)	−0.0071 (18)
C1_1	0.046 (3)	0.039 (4)	0.031 (3)	−0.002 (3)	−0.007 (3)	−0.008 (3)
N2_1	0.046 (2)	0.0370 (19)	0.0261 (17)	−0.0026 (16)	−0.0105 (16)	−0.0048 (15)
C3_1	0.045 (4)	0.042 (4)	0.027 (3)	−0.005 (3)	−0.009 (3)	−0.004 (3)
C3A_1	0.047 (3)	0.046 (3)	0.030 (2)	−0.002 (3)	−0.010 (2)	−0.007 (2)
C4_1	0.050 (3)	0.051 (4)	0.037 (3)	−0.007 (3)	−0.007 (3)	−0.014 (3)
C5_1	0.052 (3)	0.059 (4)	0.034 (3)	−0.008 (3)	−0.013 (3)	−0.014 (3)
C6_1	0.056 (3)	0.052 (3)	0.028 (3)	−0.003 (3)	−0.009 (3)	−0.005 (3)
C7_1	0.053 (3)	0.052 (3)	0.033 (3)	−0.008 (3)	−0.008 (3)	−0.011 (3)
C7A_1	0.046 (3)	0.046 (3)	0.033 (3)	−0.003 (3)	−0.009 (2)	−0.012 (2)
O1_1	0.047 (3)	0.047 (3)	0.031 (2)	0.002 (2)	−0.011 (2)	−0.006 (2)
C1B_1	0.046 (5)	0.041 (5)	0.027 (4)	−0.003 (4)	−0.007 (4)	−0.006 (4)
N2B_1	0.046 (2)	0.0370 (19)	0.0261 (17)	−0.0026 (16)	−0.0105 (16)	−0.0048 (15)
C3B_1	0.046 (5)	0.042 (5)	0.029 (4)	0.000 (4)	−0.008 (4)	−0.008 (4)
C3AB_1	0.049 (4)	0.044 (4)	0.031 (3)	−0.002 (4)	−0.010 (3)	−0.009 (3)
C4B_1	0.053 (5)	0.047 (5)	0.031 (5)	0.000 (5)	−0.008 (5)	−0.012 (4)
C5B_1	0.054 (5)	0.050 (5)	0.029 (5)	−0.003 (5)	−0.010 (5)	−0.010 (4)
C6B_1	0.051 (4)	0.051 (4)	0.030 (3)	−0.002 (4)	−0.010 (3)	−0.009 (3)
C7B_1	0.053 (4)	0.049 (4)	0.033 (4)	−0.003 (4)	−0.011 (4)	−0.010 (4)
C7AB_1	0.049 (4)	0.046 (4)	0.031 (4)	−0.002 (4)	−0.008 (4)	−0.010 (4)
O1B_1	0.050 (4)	0.045 (4)	0.031 (3)	0.002 (4)	−0.011 (3)	−0.010 (3)
C8_1	0.038 (2)	0.036 (2)	0.031 (2)	0.008 (2)	−0.013 (2)	−0.0065 (19)
C9_1	0.039 (3)	0.046 (3)	0.027 (2)	0.004 (2)	−0.008 (2)	−0.009 (2)
C10_1	0.054 (3)	0.046 (3)	0.040 (3)	0.018 (3)	−0.012 (2)	−0.015 (2)
C11_1	0.053 (3)	0.061 (4)	0.044 (3)	0.014 (3)	−0.006 (3)	−0.024 (3)
C12_1	0.048 (3)	0.070 (4)	0.030 (3)	0.007 (3)	−0.006 (2)	−0.014 (3)
C13_1	0.052 (3)	0.046 (3)	0.032 (3)	−0.001 (2)	−0.008 (2)	−0.005 (2)
C14_1	0.043 (3)	0.042 (3)	0.031 (2)	−0.001 (2)	−0.007 (2)	−0.010 (2)
Br1_2	0.0636 (4)	0.0754 (5)	0.0256 (3)	0.0077 (3)	−0.0095 (3)	−0.0158 (3)
Se1_2	0.0427 (3)	0.0443 (3)	0.0329 (3)	0.0030 (2)	−0.0072 (2)	−0.0131 (2)
N1_2	0.041 (2)	0.044 (2)	0.027 (2)	−0.0023 (18)	−0.0071 (17)	−0.0134 (17)
C1_2	0.039 (3)	0.049 (3)	0.030 (2)	−0.002 (2)	−0.007 (2)	−0.008 (2)
N2_2	0.0386 (19)	0.049 (2)	0.0271 (18)	−0.0029 (17)	−0.0078 (15)	−0.0119 (16)
C3_2	0.043 (3)	0.057 (4)	0.028 (2)	0.004 (3)	−0.008 (2)	−0.014 (2)
C3A_2	0.048 (3)	0.053 (3)	0.028 (2)	0.000 (2)	−0.010 (2)	−0.015 (2)
C4_2	0.051 (3)	0.064 (4)	0.032 (3)	0.004 (3)	−0.013 (2)	−0.019 (3)
C5_2	0.054 (3)	0.061 (3)	0.031 (3)	0.001 (3)	−0.012 (2)	−0.016 (2)
C6_2	0.054 (3)	0.061 (3)	0.028 (2)	−0.005 (3)	−0.009 (2)	−0.012 (2)
C7_2	0.047 (3)	0.059 (3)	0.029 (2)	−0.001 (3)	−0.008 (2)	−0.013 (2)
C7A_2	0.044 (3)	0.054 (3)	0.030 (2)	−0.001 (2)	−0.007 (2)	−0.014 (2)
O1_2	0.058 (3)	0.053 (2)	0.0291 (19)	−0.006 (2)	−0.0072 (18)	−0.0140 (18)
C1B_2	0.042 (5)	0.051 (5)	0.028 (5)	0.000 (5)	−0.009 (4)	−0.014 (5)
N2B_2	0.0386 (19)	0.049 (2)	0.0271 (18)	−0.0029 (17)	−0.0078 (15)	−0.0119 (16)
C3B_2	0.041 (5)	0.050 (5)	0.028 (5)	−0.003 (5)	−0.008 (5)	−0.011 (5)
C3AB_2	0.047 (4)	0.054 (4)	0.030 (4)	−0.001 (4)	−0.007 (4)	−0.012 (4)
C4B_2	0.048 (5)	0.057 (6)	0.032 (5)	−0.001 (5)	−0.008 (5)	−0.014 (5)
C5B_2	0.052 (5)	0.061 (5)	0.030 (5)	−0.001 (5)	−0.009 (5)	−0.016 (5)
C6B_2	0.052 (4)	0.059 (4)	0.029 (4)	−0.003 (4)	−0.009 (4)	−0.014 (4)
C7B_2	0.052 (4)	0.059 (4)	0.030 (4)	0.000 (4)	−0.010 (4)	−0.015 (4)
C7AB_2	0.048 (4)	0.056 (4)	0.029 (4)	0.001 (4)	−0.010 (4)	−0.014 (4)
O1B_2	0.050 (4)	0.055 (4)	0.029 (4)	−0.002 (4)	−0.007 (4)	−0.013 (4)
C8_2	0.042 (3)	0.032 (2)	0.028 (2)	−0.007 (2)	−0.006 (2)	−0.0052 (18)
C9_2	0.038 (3)	0.044 (3)	0.029 (2)	0.001 (2)	−0.008 (2)	−0.013 (2)
C10_2	0.051 (3)	0.041 (3)	0.035 (3)	−0.004 (2)	−0.012 (2)	−0.010 (2)
C11_2	0.054 (3)	0.041 (3)	0.033 (3)	0.001 (2)	−0.016 (2)	−0.014 (2)
C12_2	0.042 (3)	0.052 (3)	0.026 (2)	0.011 (2)	−0.011 (2)	−0.014 (2)
C13_2	0.046 (3)	0.055 (3)	0.032 (3)	−0.006 (3)	−0.004 (2)	−0.007 (2)
C14_2	0.042 (3)	0.048 (3)	0.034 (3)	−0.007 (2)	−0.008 (2)	−0.013 (2)

### (3aRS,6RS,7aRS)-N-(4-Bromophenyl)-1,6,7,7a-tetrahydro-3a,6-epoxyisoindole-2(3H)-carboselenoamide Geometric parameters (Å, º)

**Table:**

Br1_1—C12_1	1.908 (6)	Br1_2—C12_2	1.903 (5)
Se1_1—C8_1	1.862 (6)	Se1_2—C8_2	1.873 (5)
N1_1—C8_1	1.349 (7)	N1_2—C8_2	1.342 (7)
N1_1—C9_1	1.421 (6)	N1_2—C9_2	1.434 (6)
N1_1—H1_1	0.8800	N1_2—H1_2	0.8800
C1_1—N2_1	1.477 (10)	C1_2—N2_2	1.487 (9)
C1_1—C7A_1	1.528 (11)	C1_2—C7A_2	1.532 (10)
C1_1—H1A_1	0.9900	C1_2—H1A_2	0.9900
C1_1—H1B_1	0.9900	C1_2—H1B_2	0.9900
N2_1—C8_1	1.334 (7)	N2_2—C8_2	1.329 (6)
N2_1—C3_1	1.475 (11)	N2_2—C3_2	1.475 (8)
C3_1—C3A_1	1.505 (11)	C3_2—C3A_2	1.511 (9)
C3_1—H3A_1	0.9900	C3_2—H3A_2	0.9900
C3_1—H3B_1	0.9900	C3_2—H3B_2	0.9900
C3A_1—O1_1	1.455 (9)	C3A_2—O1_2	1.446 (9)
C3A_1—C4_1	1.503 (10)	C3A_2—C4_2	1.515 (9)
C3A_1—C7A_1	1.557 (10)	C3A_2—C7A_2	1.556 (9)
C4_1—C5_1	1.331 (11)	C4_2—C5_2	1.314 (10)
C4_1—H4_1	0.9500	C4_2—H4_2	0.9500
C5_1—C6_1	1.506 (12)	C5_2—C6_2	1.506 (10)
C5_1—H5_1	0.9500	C5_2—H5_2	0.9500
C6_1—O1_1	1.440 (9)	C6_2—O1_2	1.446 (8)
C6_1—C7_1	1.556 (12)	C6_2—C7_2	1.552 (10)
C6_1—H6_1	1.0000	C6_2—H6_2	1.0000
C7_1—C7A_1	1.540 (10)	C7_2—C7A_2	1.542 (9)
C7_1—H7A_1	0.9900	C7_2—H7A_2	0.9900
C7_1—H7B_1	0.9900	C7_2—H7B_2	0.9900
C7A_1—H7AA_1	1.0000	C7A_2—H7AA_2	1.0000
C1B_1—N2B_1	1.474 (18)	C1B_2—N2B_2	1.474 (18)
C1B_1—C7AB_1	1.521 (18)	C1B_2—C7AB_2	1.533 (19)
C1B_1—H1C_1	0.9900	C1B_2—H1C_2	0.9900
C1B_1—H1D_1	0.9900	C1B_2—H1D_2	0.9900
N2B_1—C8_1	1.334 (7)	N2B_2—C8_2	1.329 (6)
N2B_1—C3B_1	1.466 (17)	N2B_2—C3B_2	1.454 (18)
C3B_1—C3AB_1	1.504 (17)	C3B_2—C3AB_2	1.504 (18)
C3B_1—H3C_1	0.9900	C3B_2—H3C_2	0.9900
C3B_1—H3D_1	0.9900	C3B_2—H3D_2	0.9900
C3AB_1—O1B_1	1.441 (15)	C3AB_2—O1B_2	1.443 (17)
C3AB_1—C4B_1	1.497 (15)	C3AB_2—C4B_2	1.498 (16)
C3AB_1—C7AB_1	1.561 (15)	C3AB_2—C7AB_2	1.561 (16)
C4B_1—C5B_1	1.314 (16)	C4B_2—C5B_2	1.309 (17)
C4B_1—H4B_1	0.9500	C4B_2—H4B_2	0.9500
C5B_1—C6B_1	1.512 (17)	C5B_2—C6B_2	1.513 (18)
C5B_1—H5B_1	0.9500	C5B_2—H5B_2	0.9500
C6B_1—O1B_1	1.457 (15)	C6B_2—O1B_2	1.448 (16)
C6B_1—C7B_1	1.549 (17)	C6B_2—C7B_2	1.550 (19)
C6B_1—H6B_1	1.0000	C6B_2—H6B_2	1.0000
C7B_1—C7AB_1	1.555 (16)	C7B_2—C7AB_2	1.552 (17)
C7B_1—H7C_1	0.9900	C7B_2—H7C_2	0.9900
C7B_1—H7D_1	0.9900	C7B_2—H7D_2	0.9900
C7AB_1—H7AB_1	1.0000	C7AB_2—H7AB_2	1.0000
C9_1—C14_1	1.387 (8)	C9_2—C10_2	1.381 (8)
C9_1—C10_1	1.396 (8)	C9_2—C14_2	1.390 (8)
C10_1—C11_1	1.375 (8)	C10_2—C11_2	1.394 (8)
C10_1—H10_1	0.9500	C10_2—H10_2	0.9500
C11_1—C12_1	1.389 (10)	C11_2—C12_2	1.383 (9)
C11_1—H11_1	0.9500	C11_2—H11_2	0.9500
C12_1—C13_1	1.371 (9)	C12_2—C13_2	1.392 (9)
C13_1—C14_1	1.385 (7)	C13_2—C14_2	1.385 (8)
C13_1—H13_1	0.9500	C13_2—H13_2	0.9500
C14_1—H14_1	0.9500	C14_2—H14_2	0.9500
			
C8_1—N1_1—C9_1	126.4 (5)	C8_2—N1_2—C9_2	123.3 (4)
C8_1—N1_1—H1_1	116.8	C8_2—N1_2—H1_2	118.4
C9_1—N1_1—H1_1	116.8	C9_2—N1_2—H1_2	118.4
N2_1—C1_1—C7A_1	105.4 (7)	N2_2—C1_2—C7A_2	104.9 (6)
N2_1—C1_1—H1A_1	110.7	N2_2—C1_2—H1A_2	110.8
C7A_1—C1_1—H1A_1	110.7	C7A_2—C1_2—H1A_2	110.8
N2_1—C1_1—H1B_1	110.7	N2_2—C1_2—H1B_2	110.8
C7A_1—C1_1—H1B_1	110.7	C7A_2—C1_2—H1B_2	110.8
H1A_1—C1_1—H1B_1	108.8	H1A_2—C1_2—H1B_2	108.8
C8_1—N2_1—C3_1	123.8 (5)	C8_2—N2_2—C3_2	123.7 (5)
C8_1—N2_1—C1_1	124.7 (6)	C8_2—N2_2—C1_2	123.8 (5)
C3_1—N2_1—C1_1	111.4 (5)	C3_2—N2_2—C1_2	112.4 (5)
N2_1—C3_1—C3A_1	104.8 (7)	N2_2—C3_2—C3A_2	103.1 (6)
N2_1—C3_1—H3A_1	110.8	N2_2—C3_2—H3A_2	111.1
C3A_1—C3_1—H3A_1	110.8	C3A_2—C3_2—H3A_2	111.1
N2_1—C3_1—H3B_1	110.8	N2_2—C3_2—H3B_2	111.1
C3A_1—C3_1—H3B_1	110.8	C3A_2—C3_2—H3B_2	111.1
H3A_1—C3_1—H3B_1	108.9	H3A_2—C3_2—H3B_2	109.1
O1_1—C3A_1—C4_1	101.3 (6)	O1_2—C3A_2—C3_2	112.7 (6)
O1_1—C3A_1—C3_1	112.4 (7)	O1_2—C3A_2—C4_2	100.9 (5)
C4_1—C3A_1—C3_1	125.2 (7)	C3_2—C3A_2—C4_2	125.0 (6)
O1_1—C3A_1—C7A_1	99.3 (6)	O1_2—C3A_2—C7A_2	99.7 (5)
C4_1—C3A_1—C7A_1	109.4 (7)	C3_2—C3A_2—C7A_2	106.7 (5)
C3_1—C3A_1—C7A_1	106.2 (6)	C4_2—C3A_2—C7A_2	109.0 (6)
C5_1—C4_1—C3A_1	105.2 (7)	C5_2—C4_2—C3A_2	106.1 (6)
C5_1—C4_1—H4_1	127.4	C5_2—C4_2—H4_2	127.0
C3A_1—C4_1—H4_1	127.4	C3A_2—C4_2—H4_2	127.0
C4_1—C5_1—C6_1	106.1 (7)	C4_2—C5_2—C6_2	105.5 (6)
C4_1—C5_1—H5_1	126.9	C4_2—C5_2—H5_2	127.2
C6_1—C5_1—H5_1	126.9	C6_2—C5_2—H5_2	127.2
O1_1—C6_1—C5_1	101.7 (7)	O1_2—C6_2—C5_2	101.3 (6)
O1_1—C6_1—C7_1	100.6 (6)	O1_2—C6_2—C7_2	101.0 (5)
C5_1—C6_1—C7_1	108.0 (7)	C5_2—C6_2—C7_2	109.0 (7)
O1_1—C6_1—H6_1	115.0	O1_2—C6_2—H6_2	114.6
C5_1—C6_1—H6_1	115.0	C5_2—C6_2—H6_2	114.6
C7_1—C6_1—H6_1	115.0	C7_2—C6_2—H6_2	114.6
C7A_1—C7_1—C6_1	100.5 (6)	C7A_2—C7_2—C6_2	100.5 (6)
C7A_1—C7_1—H7A_1	111.7	C7A_2—C7_2—H7A_2	111.7
C6_1—C7_1—H7A_1	111.7	C6_2—C7_2—H7A_2	111.7
C7A_1—C7_1—H7B_1	111.7	C7A_2—C7_2—H7B_2	111.7
C6_1—C7_1—H7B_1	111.7	C6_2—C7_2—H7B_2	111.7
H7A_1—C7_1—H7B_1	109.4	H7A_2—C7_2—H7B_2	109.4
C1_1—C7A_1—C7_1	117.9 (7)	C1_2—C7A_2—C7_2	117.3 (7)
C1_1—C7A_1—C3A_1	102.5 (6)	C1_2—C7A_2—C3A_2	101.8 (6)
C7_1—C7A_1—C3A_1	101.8 (6)	C7_2—C7A_2—C3A_2	101.6 (5)
C1_1—C7A_1—H7AA_1	111.3	C1_2—C7A_2—H7AA_2	111.7
C7_1—C7A_1—H7AA_1	111.3	C7_2—C7A_2—H7AA_2	111.7
C3A_1—C7A_1—H7AA_1	111.3	C3A_2—C7A_2—H7AA_2	111.7
C6_1—O1_1—C3A_1	95.5 (5)	C6_2—O1_2—C3A_2	95.2 (5)
N2B_1—C1B_1—C7AB_1	101.4 (13)	N2B_2—C1B_2—C7AB_2	99.9 (14)
N2B_1—C1B_1—H1C_1	111.5	N2B_2—C1B_2—H1C_2	111.8
C7AB_1—C1B_1—H1C_1	111.5	C7AB_2—C1B_2—H1C_2	111.8
N2B_1—C1B_1—H1D_1	111.5	N2B_2—C1B_2—H1D_2	111.8
C7AB_1—C1B_1—H1D_1	111.5	C7AB_2—C1B_2—H1D_2	111.8
H1C_1—C1B_1—H1D_1	109.3	H1C_2—C1B_2—H1D_2	109.5
C8_1—N2B_1—C3B_1	119.5 (8)	C8_2—N2B_2—C3B_2	122.3 (10)
C8_1—N2B_1—C1B_1	125.0 (9)	C8_2—N2B_2—C1B_2	119.6 (10)
C3B_1—N2B_1—C1B_1	115.5 (10)	C3B_2—N2B_2—C1B_2	117.3 (12)
N2B_1—C3B_1—C3AB_1	101.6 (12)	N2B_2—C3B_2—C3AB_2	99.2 (13)
N2B_1—C3B_1—H3C_1	111.4	N2B_2—C3B_2—H3C_2	111.9
C3AB_1—C3B_1—H3C_1	111.4	C3AB_2—C3B_2—H3C_2	111.9
N2B_1—C3B_1—H3D_1	111.4	N2B_2—C3B_2—H3D_2	111.9
C3AB_1—C3B_1—H3D_1	111.4	C3AB_2—C3B_2—H3D_2	111.9
H3C_1—C3B_1—H3D_1	109.3	H3C_2—C3B_2—H3D_2	109.6
O1B_1—C3AB_1—C4B_1	102.2 (12)	O1B_2—C3AB_2—C4B_2	102.1 (14)
O1B_1—C3AB_1—C3B_1	114.2 (16)	O1B_2—C3AB_2—C3B_2	113 (2)
C4B_1—C3AB_1—C3B_1	123.5 (14)	C4B_2—C3AB_2—C3B_2	121.6 (18)
O1B_1—C3AB_1—C7AB_1	99.5 (12)	O1B_2—C3AB_2—C7AB_2	98.6 (14)
C4B_1—C3AB_1—C7AB_1	108.3 (13)	C4B_2—C3AB_2—C7AB_2	109.8 (17)
C3B_1—C3AB_1—C7AB_1	106.6 (12)	C3B_2—C3AB_2—C7AB_2	109.4 (15)
C5B_1—C4B_1—C3AB_1	105.9 (13)	C5B_2—C4B_2—C3AB_2	104.4 (14)
C5B_1—C4B_1—H4B_1	127.0	C5B_2—C4B_2—H4B_2	127.8
C3AB_1—C4B_1—H4B_1	127.0	C3AB_2—C4B_2—H4B_2	127.8
C4B_1—C5B_1—C6B_1	106.1 (13)	C4B_2—C5B_2—C6B_2	107.1 (15)
C4B_1—C5B_1—H5B_1	126.9	C4B_2—C5B_2—H5B_2	126.5
C6B_1—C5B_1—H5B_1	126.9	C6B_2—C5B_2—H5B_2	126.5
O1B_1—C6B_1—C5B_1	100.9 (13)	O1B_2—C6B_2—C5B_2	102.7 (15)
O1B_1—C6B_1—C7B_1	100.8 (13)	O1B_2—C6B_2—C7B_2	99.0 (15)
C5B_1—C6B_1—C7B_1	107.6 (16)	C5B_2—C6B_2—C7B_2	107.4 (18)
O1B_1—C6B_1—H6B_1	115.2	O1B_2—C6B_2—H6B_2	115.3
C5B_1—C6B_1—H6B_1	115.2	C5B_2—C6B_2—H6B_2	115.3
C7B_1—C6B_1—H6B_1	115.2	C7B_2—C6B_2—H6B_2	115.3
C6B_1—C7B_1—C7AB_1	100.6 (12)	C6B_2—C7B_2—C7AB_2	99.5 (13)
C6B_1—C7B_1—H7C_1	111.7	C6B_2—C7B_2—H7C_2	111.9
C7AB_1—C7B_1—H7C_1	111.7	C7AB_2—C7B_2—H7C_2	111.9
C6B_1—C7B_1—H7D_1	111.7	C6B_2—C7B_2—H7D_2	111.9
C7AB_1—C7B_1—H7D_1	111.7	C7AB_2—C7B_2—H7D_2	111.9
H7C_1—C7B_1—H7D_1	109.4	H7C_2—C7B_2—H7D_2	109.6
C1B_1—C7AB_1—C7B_1	118.0 (17)	C1B_2—C7AB_2—C7B_2	115 (2)
C1B_1—C7AB_1—C3AB_1	102.8 (13)	C1B_2—C7AB_2—C3AB_2	100.8 (15)
C7B_1—C7AB_1—C3AB_1	101.3 (11)	C7B_2—C7AB_2—C3AB_2	102.1 (13)
C1B_1—C7AB_1—H7AB_1	111.3	C1B_2—C7AB_2—H7AB_2	112.7
C7B_1—C7AB_1—H7AB_1	111.3	C7B_2—C7AB_2—H7AB_2	112.7
C3AB_1—C7AB_1—H7AB_1	111.3	C3AB_2—C7AB_2—H7AB_2	112.7
C3AB_1—O1B_1—C6B_1	95.5 (11)	C3AB_2—O1B_2—C6B_2	95.4 (12)
N2B_1—C8_1—N1_1	117.0 (5)	N2B_2—C8_2—N1_2	118.0 (5)
N2_1—C8_1—N1_1	117.0 (5)	N2_2—C8_2—N1_2	118.0 (5)
N2B_1—C8_1—Se1_1	121.1 (4)	N2B_2—C8_2—Se1_2	121.4 (4)
N2_1—C8_1—Se1_1	121.1 (4)	N2_2—C8_2—Se1_2	121.4 (4)
N1_1—C8_1—Se1_1	121.9 (4)	N1_2—C8_2—Se1_2	120.5 (4)
C14_1—C9_1—C10_1	119.8 (5)	C10_2—C9_2—C14_2	121.3 (5)
C14_1—C9_1—N1_1	118.6 (5)	C10_2—C9_2—N1_2	119.3 (5)
C10_1—C9_1—N1_1	121.5 (5)	C14_2—C9_2—N1_2	119.4 (5)
C11_1—C10_1—C9_1	120.0 (5)	C9_2—C10_2—C11_2	119.3 (5)
C11_1—C10_1—H10_1	120.0	C9_2—C10_2—H10_2	120.3
C9_1—C10_1—H10_1	120.0	C11_2—C10_2—H10_2	120.3
C10_1—C11_1—C12_1	119.1 (6)	C12_2—C11_2—C10_2	119.0 (5)
C10_1—C11_1—H11_1	120.4	C12_2—C11_2—H11_2	120.5
C12_1—C11_1—H11_1	120.4	C10_2—C11_2—H11_2	120.5
C13_1—C12_1—C11_1	121.8 (5)	C11_2—C12_2—C13_2	122.0 (5)
C13_1—C12_1—Br1_1	118.8 (5)	C11_2—C12_2—Br1_2	118.5 (4)
C11_1—C12_1—Br1_1	119.4 (5)	C13_2—C12_2—Br1_2	119.5 (4)
C12_1—C13_1—C14_1	118.9 (5)	C14_2—C13_2—C12_2	118.6 (5)
C12_1—C13_1—H13_1	120.5	C14_2—C13_2—H13_2	120.7
C14_1—C13_1—H13_1	120.5	C12_2—C13_2—H13_2	120.7
C13_1—C14_1—C9_1	120.4 (5)	C13_2—C14_2—C9_2	119.8 (5)
C13_1—C14_1—H14_1	119.8	C13_2—C14_2—H14_2	120.1
C9_1—C14_1—H14_1	119.8	C9_2—C14_2—H14_2	120.1
			
C7A_1—C1_1—N2_1—C8_1	163.1 (6)	C7A_2—C1_2—N2_2—C8_2	163.4 (6)
C7A_1—C1_1—N2_1—C3_1	−14.2 (9)	C7A_2—C1_2—N2_2—C3_2	−11.5 (11)
C8_1—N2_1—C3_1—C3A_1	177.0 (6)	C8_2—N2_2—C3_2—C3A_2	175.4 (5)
C1_1—N2_1—C3_1—C3A_1	−5.7 (10)	C1_2—N2_2—C3_2—C3A_2	−9.8 (9)
N2_1—C3_1—C3A_1—O1_1	−84.5 (9)	N2_2—C3_2—C3A_2—O1_2	−81.3 (7)
N2_1—C3_1—C3A_1—C4_1	152.1 (8)	N2_2—C3_2—C3A_2—C4_2	155.8 (7)
N2_1—C3_1—C3A_1—C7A_1	23.0 (10)	N2_2—C3_2—C3A_2—C7A_2	27.1 (8)
O1_1—C3A_1—C4_1—C5_1	34.3 (9)	O1_2—C3A_2—C4_2—C5_2	32.5 (8)
C3_1—C3A_1—C4_1—C5_1	162.4 (9)	C3_2—C3A_2—C4_2—C5_2	160.4 (7)
C7A_1—C3A_1—C4_1—C5_1	−69.9 (9)	C7A_2—C3A_2—C4_2—C5_2	−71.8 (8)
C3A_1—C4_1—C5_1—C6_1	−2.2 (10)	C3A_2—C4_2—C5_2—C6_2	0.7 (9)
C4_1—C5_1—C6_1—O1_1	−31.0 (9)	C4_2—C5_2—C6_2—O1_2	−33.7 (8)
C4_1—C5_1—C6_1—C7_1	74.3 (9)	C4_2—C5_2—C6_2—C7_2	72.3 (8)
O1_1—C6_1—C7_1—C7A_1	36.7 (8)	O1_2—C6_2—C7_2—C7A_2	35.7 (7)
C5_1—C6_1—C7_1—C7A_1	−69.4 (8)	C5_2—C6_2—C7_2—C7A_2	−70.5 (7)
N2_1—C1_1—C7A_1—C7_1	137.9 (7)	N2_2—C1_2—C7A_2—C7_2	136.7 (7)
N2_1—C1_1—C7A_1—C3A_1	27.2 (8)	N2_2—C1_2—C7A_2—C3A_2	26.9 (10)
C6_1—C7_1—C7A_1—C1_1	−110.7 (8)	C6_2—C7_2—C7A_2—C1_2	−108.5 (8)
C6_1—C7_1—C7A_1—C3A_1	0.5 (8)	C6_2—C7_2—C7A_2—C3A_2	1.4 (7)
O1_1—C3A_1—C7A_1—C1_1	85.4 (7)	O1_2—C3A_2—C7A_2—C1_2	83.4 (7)
C4_1—C3A_1—C7A_1—C1_1	−169.0 (7)	C3_2—C3A_2—C7A_2—C1_2	−34.0 (9)
C3_1—C3A_1—C7A_1—C1_1	−31.2 (9)	C4_2—C3A_2—C7A_2—C1_2	−171.4 (7)
O1_1—C3A_1—C7A_1—C7_1	−36.9 (7)	O1_2—C3A_2—C7A_2—C7_2	−37.9 (6)
C4_1—C3A_1—C7A_1—C7_1	68.7 (8)	C3_2—C3A_2—C7A_2—C7_2	−155.3 (6)
C3_1—C3A_1—C7A_1—C7_1	−153.6 (8)	C4_2—C3A_2—C7A_2—C7_2	67.2 (7)
C5_1—C6_1—O1_1—C3A_1	49.9 (7)	C5_2—C6_2—O1_2—C3A_2	51.6 (6)
C7_1—C6_1—O1_1—C3A_1	−61.2 (7)	C7_2—C6_2—O1_2—C3A_2	−60.6 (6)
C4_1—C3A_1—O1_1—C6_1	−51.4 (7)	C3_2—C3A_2—O1_2—C6_2	173.7 (6)
C3_1—C3A_1—O1_1—C6_1	172.7 (7)	C4_2—C3A_2—O1_2—C6_2	−50.8 (6)
C7A_1—C3A_1—O1_1—C6_1	60.7 (7)	C7A_2—C3A_2—O1_2—C6_2	60.8 (6)
C7AB_1—C1B_1—N2B_1—C8_1	−158.9 (12)	C7AB_2—C1B_2—N2B_2—C8_2	−162.8 (15)
C7AB_1—C1B_1—N2B_1—C3B_1	20 (3)	C7AB_2—C1B_2—N2B_2—C3B_2	27 (4)
C8_1—N2B_1—C3B_1—C3AB_1	−179.5 (11)	C8_2—N2B_2—C3B_2—C3AB_2	−176.0 (14)
C1B_1—N2B_1—C3B_1—C3AB_1	2 (3)	C1B_2—N2B_2—C3B_2—C3AB_2	−6 (4)
N2B_1—C3B_1—C3AB_1—O1B_1	86.4 (19)	N2B_2—C3B_2—C3AB_2—O1B_2	91 (3)
N2B_1—C3B_1—C3AB_1—C4B_1	−148.5 (17)	N2B_2—C3B_2—C3AB_2—C4B_2	−147 (3)
N2B_1—C3B_1—C3AB_1—C7AB_1	−22 (2)	N2B_2—C3B_2—C3AB_2—C7AB_2	−18 (4)
O1B_1—C3AB_1—C4B_1—C5B_1	−32.3 (19)	O1B_2—C3AB_2—C4B_2—C5B_2	−39 (3)
C3B_1—C3AB_1—C4B_1—C5B_1	−162 (2)	C3B_2—C3AB_2—C4B_2—C5B_2	−166 (3)
C7AB_1—C3AB_1—C4B_1—C5B_1	72.2 (19)	C7AB_2—C3AB_2—C4B_2—C5B_2	65 (3)
C3AB_1—C4B_1—C5B_1—C6B_1	0 (2)	C3AB_2—C4B_2—C5B_2—C6B_2	9 (3)
C4B_1—C5B_1—C6B_1—O1B_1	32.4 (19)	C4B_2—C5B_2—C6B_2—O1B_2	24 (3)
C4B_1—C5B_1—C6B_1—C7B_1	−72.8 (19)	C4B_2—C5B_2—C6B_2—C7B_2	−80 (3)
O1B_1—C6B_1—C7B_1—C7AB_1	−35.3 (19)	O1B_2—C6B_2—C7B_2—C7AB_2	−41 (2)
C5B_1—C6B_1—C7B_1—C7AB_1	70.0 (18)	C5B_2—C6B_2—C7B_2—C7AB_2	66 (2)
N2B_1—C1B_1—C7AB_1—C7B_1	−141.7 (18)	N2B_2—C1B_2—C7AB_2—C7B_2	−143 (2)
N2B_1—C1B_1—C7AB_1—C3AB_1	−31 (2)	N2B_2—C1B_2—C7AB_2—C3AB_2	−34 (3)
C6B_1—C7B_1—C7AB_1—C1B_1	110 (2)	C6B_2—C7B_2—C7AB_2—C1B_2	112 (3)
C6B_1—C7B_1—C7AB_1—C3AB_1	−1.6 (19)	C6B_2—C7B_2—C7AB_2—C3AB_2	4 (2)
O1B_1—C3AB_1—C7AB_1—C1B_1	−84.0 (18)	O1B_2—C3AB_2—C7AB_2—C1B_2	−84 (2)
C4B_1—C3AB_1—C7AB_1—C1B_1	169.6 (18)	C4B_2—C3AB_2—C7AB_2—C1B_2	170 (2)
C3B_1—C3AB_1—C7AB_1—C1B_1	35 (2)	C3B_2—C3AB_2—C7AB_2—C1B_2	34 (3)
O1B_1—C3AB_1—C7AB_1—C7B_1	38.3 (16)	O1B_2—C3AB_2—C7AB_2—C7B_2	34 (2)
C4B_1—C3AB_1—C7AB_1—C7B_1	−68.0 (17)	C4B_2—C3AB_2—C7AB_2—C7B_2	−72 (2)
C3B_1—C3AB_1—C7AB_1—C7B_1	157.2 (18)	C3B_2—C3AB_2—C7AB_2—C7B_2	152 (3)
C4B_1—C3AB_1—O1B_1—C6B_1	50.0 (15)	C4B_2—C3AB_2—O1B_2—C6B_2	51.3 (18)
C3B_1—C3AB_1—O1B_1—C6B_1	−174.3 (13)	C3B_2—C3AB_2—O1B_2—C6B_2	−176.6 (16)
C7AB_1—C3AB_1—O1B_1—C6B_1	−61.2 (13)	C7AB_2—C3AB_2—O1B_2—C6B_2	−61.2 (16)
C5B_1—C6B_1—O1B_1—C3AB_1	−49.7 (14)	C5B_2—C6B_2—O1B_2—C3AB_2	−45.4 (19)
C7B_1—C6B_1—O1B_1—C3AB_1	60.8 (16)	C7B_2—C6B_2—O1B_2—C3AB_2	64.8 (17)
C3B_1—N2B_1—C8_1—N1_1	174.7 (15)	C3B_2—N2B_2—C8_2—N1_2	−1 (3)
C1B_1—N2B_1—C8_1—N1_1	−7 (2)	C1B_2—N2B_2—C8_2—N1_2	−170 (2)
C3B_1—N2B_1—C8_1—Se1_1	−2.7 (16)	C3B_2—N2B_2—C8_2—Se1_2	177 (3)
C1B_1—N2B_1—C8_1—Se1_1	175.8 (19)	C1B_2—N2B_2—C8_2—Se1_2	8 (2)
C3_1—N2_1—C8_1—N1_1	3.4 (9)	C3_2—N2_2—C8_2—N1_2	174.4 (6)
C1_1—N2_1—C8_1—N1_1	−173.5 (6)	C1_2—N2_2—C8_2—N1_2	0.1 (10)
C3_1—N2_1—C8_1—Se1_1	−174.0 (7)	C3_2—N2_2—C8_2—Se1_2	−7.5 (8)
C1_1—N2_1—C8_1—Se1_1	9.1 (8)	C1_2—N2_2—C8_2—Se1_2	178.2 (7)
C9_1—N1_1—C8_1—N2B_1	171.6 (5)	C9_2—N1_2—C8_2—N2B_2	−175.2 (5)
C9_1—N1_1—C8_1—N2_1	171.6 (5)	C9_2—N1_2—C8_2—N2_2	−175.2 (5)
C9_1—N1_1—C8_1—Se1_1	−11.0 (7)	C9_2—N1_2—C8_2—Se1_2	6.7 (7)
C8_1—N1_1—C9_1—C14_1	−115.1 (6)	C8_2—N1_2—C9_2—C10_2	−98.1 (6)
C8_1—N1_1—C9_1—C10_1	67.9 (8)	C8_2—N1_2—C9_2—C14_2	83.8 (7)
C14_1—C9_1—C10_1—C11_1	−2.0 (9)	C14_2—C9_2—C10_2—C11_2	−1.7 (8)
N1_1—C9_1—C10_1—C11_1	174.9 (6)	N1_2—C9_2—C10_2—C11_2	−179.8 (5)
C9_1—C10_1—C11_1—C12_1	0.5 (10)	C9_2—C10_2—C11_2—C12_2	1.1 (8)
C10_1—C11_1—C12_1—C13_1	0.6 (11)	C10_2—C11_2—C12_2—C13_2	−0.2 (8)
C10_1—C11_1—C12_1—Br1_1	−177.9 (5)	C10_2—C11_2—C12_2—Br1_2	−179.4 (4)
C11_1—C12_1—C13_1—C14_1	−0.2 (10)	C11_2—C12_2—C13_2—C14_2	−0.1 (9)
Br1_1—C12_1—C13_1—C14_1	178.4 (5)	Br1_2—C12_2—C13_2—C14_2	179.0 (4)
C12_1—C13_1—C14_1—C9_1	−1.4 (9)	C12_2—C13_2—C14_2—C9_2	−0.4 (9)
C10_1—C9_1—C14_1—C13_1	2.5 (9)	C10_2—C9_2—C14_2—C13_2	1.4 (9)
N1_1—C9_1—C14_1—C13_1	−174.5 (5)	N1_2—C9_2—C14_2—C13_2	179.4 (5)

### (3aRS,6RS,7aRS)-N-(4-Bromophenyl)-1,6,7,7a-tetrahydro-3a,6-epoxyisoindole-2(3H)-carboselenoamide Hydrogen-bond geometry (Å, º)

*Cg*8 and *Cg*17 are the centroids of the C9_1–C14_1 and C9_2–C14_2 rings, respectively.

**Table:**

*D*—H···*A*	*D*—H	H···*A*	*D*···*A*	*D*—H···*A*
N1_1—H1_1···Se1_2^i^	0.88	2.67	3.460 (5)	151
C11_1—H11_1···O1_1^i^	0.95	2.34	3.283 (8)	174
C11_1—H11_1···O1*B*_1^i^	0.95	2.25	3.176 (14)	165
N1_2—H1_2···Se1_1^ii^	0.88	2.64	3.393 (5)	144
C11_2—H11_2···O1_2^iii^	0.95	2.44	3.368 (7)	167
C11_2—H11_2···O1*B*_2^iii^	0.95	2.48	3.36 (2)	154
C1_1—H1*A*_1···*Cg*17^ii^	0.99	2.83	3.720 (11)	150
C3_2—H3*B*_2···*Cg*8^i^	0.99	2.81	3.724 (9)	154

Symmetry codes: (i) −*x*, −*y*+1, −*z*+1; (ii) −*x*+1, −*y*+1, −*z*+1; (iii) −*x*+1, −*y*+2, −*z*+1.
