# Supplementary material for: Synthesis and crystal structure analysis of (3aRS,6RS,7aRS)-N-(4-bromo­phen­yl)-1,6,7,7a-tetra­hydro-3a,6-ep­oxy­iso­indole-2(3H)-carboseleno­amide
Source: Acta Crystallogr E Crystallogr Commun. 2026 May 7;82(Pt 6):572–7. doi: 10.1107/S2056989026004299 (PMC13239016; doi:10.1107/S2056989026004299)

checkCIF/PLATON report

Structure factors have been supplied for datablock(s) 1

THIS REPORT IS FOR GUIDANCE ONLY. IF USED AS PART OF A REVIEW PROCEDURE FOR PUBLICATION, IT SHOULD NOT REPLACE THE EXPERTISE OF AN EXPERIENCED CRYSTALLOGRAPHIC REFEREE.

No syntax errors found.   CIF dictionary   Interpreting this report

Datablock: 1

```
Bond precision:   C-C = 0.0086 A          Wavelength=1.54184

Cell:            a=9.7367(4)        b=10.3981(4)       c=15.7685(5)
                 alpha=73.059(3)    beta=76.870(3)     gamma=84.140(4)
Temperature:     100 K

                  Calculated                  Reported
Volume            1486.15(10)                 1486.15(10)
Space group       P -1                        P -1
Hall group        -P 1                        -P 1
Moiety formula    C15 H15 Br N2 O Se          C15 H15 Br N2 O Se
Sum formula       C15 H15 Br N2 O Se          C15 H15 Br N2 O Se
Mr                398.15                      398.15
Dx,g cm-3         1.780                       1.780
Z                 4                           4
Mu (mm-1)         6.543                       6.543
F000              784.0                       784.0
F000'             779.55
h,k,lmax          12,13,20                    12,13,20
Nref              6518                        6210
Tmin,Tmax         0.656,0.822                 0.353,1.000
Tmin'             0.134

Correction method= # Reported T Limits: Tmin=0.353 Tmax=1.000
AbsCorr = GAUSSIAN
```

Data completeness= 0.953 Theta(max)= 80.380

R(reflections)= 0.0699(5303) wR2(reflections)= 0.1781(6210)

S = 1.072 Npar= 525

The following ALERTS were generated. Each ALERT has the format
test-name_ALERT_alert-type_alert-level.
Click on the hyperlinks for more details of the test.

Alert level C

```
PLAT341_ALERT_3_C Low Bond Precision on  C-C Bonds ...............    0.00858 Ang.
PLAT906_ALERT_3_C Large K Value in the Analysis of Variance ......     27.556 Check
PLAT906_ALERT_3_C Large K Value in the Analysis of Variance ......      5.728 Check
PLAT911_ALERT_3_C Missing FCF Refl Between Thmin & STh/L=    0.600         36 Report
               10  6  0, -10 -5  1,    9  7  1, -10 -4  2,    9  8  2,    7  9  2,
                7 10  2,    2-11  3, -10 -4  3,    7 10  3,    6 11  3,    3-10  4,
                7  9  4,    8  9  4,    7 10  4,    6 11  4,    9  8  5,    8  9  5,
                7 10  5,    6 11  5,   -4 -4  6,    7 10  6,   -2 -9  7,    6 11  7,
               -1 -9  8,   -3 -7  8,    6 11  8,   -2 -8  9,   -3  9 10,   -3  9 11,
                   (    6 More NOT listed: see .ckf listing file)
PLAT977_ALERT_2_C Check Negative Difference Density on H1_1      .      -0.31 eA-3
```

Alert level G

```
PLAT002_ALERT_2_G Number of Distance or Angle Restraints on AtSite         40 Note
PLAT003_ALERT_2_G Number of Uiso or U(i,j)  Restrained non-H-Atoms         40 Report
PLAT007_ALERT_5_G Number of Unrefined Donor-H Atoms .............           2 Report
              H1#     H26#
PLAT168_ALERT_4_G The CIF-Embedded .res File Contains EXYZ Records          2 Report
PLAT171_ALERT_4_G The CIF-Embedded .res File Contains EADP Records          2 Report
PLAT175_ALERT_4_G The CIF-Embedded .res File Contains SAME Records          3 Report
PLAT178_ALERT_4_G The CIF-Embedded .res File Contains SIMU Records          2 Report
PLAT301_ALERT_3_G Main Residue  Disorder .............(Resd    1)         50% Note
PLAT301_ALERT_3_G Main Residue  Disorder .............(Resd    2)         50% Note
PLAT398_ALERT_2_G Deviating  C-O-C    Angle From 120 for O1_1     .      95.5 Degree
PLAT398_ALERT_2_G Deviating  C-O-C    Angle From 120 for O1B_1    .      95.4 Degree
PLAT398_ALERT_2_G Deviating  C-O-C    Angle From 120 for O1_2     .      95.2 Degree
PLAT398_ALERT_2_G Deviating  C-O-C    Angle From 120 for O1B_2    .      95.3 Degree
PLAT414_ALERT_2_G Short Intra D-H..H-X       H1_1     ..H1D_1    .       2.13 Ang.
                                                        x,y,z  =      1_555 Check
PLAT414_ALERT_2_G Short Intra D-H..H-X       H1_2     ..H1A_2    .       2.07 Ang.
                                                        x,y,z  =      1_555 Check
PLAT434_ALERT_2_G Short Inter HL..HL Contact Br1_1    ..Br1_2    .       3.59 Ang.
                                                   -x,1-y,2-z  =      2_567 Check
PLAT793_ALERT_4_G Model has Chirality at C3A_1       (Centro SpGr)          S Verify
PLAT793_ALERT_4_G Model has Chirality at C6_1        (Centro SpGr)          S Verify
PLAT793_ALERT_4_G Model has Chirality at C7A_1       (Centro SpGr)          S Verify
PLAT793_ALERT_4_G Model has Chirality at C3A_2       (Centro SpGr)          S Verify
PLAT793_ALERT_4_G Model has Chirality at C6_2        (Centro SpGr)          S Verify
PLAT793_ALERT_4_G Model has Chirality at C7A_2       (Centro SpGr)          S Verify
PLAT811_ALERT_5_G No ADDSYM Analysis: Too Many Excluded Atoms ....         40 Info
PLAT860_ALERT_3_G Number of Least-Squares Restraints ............         930 Note
```

```
PLAT912_ALERT_4_G Missing # of FCF Reflections Above STh/L=  0.600        272 Note
PLAT933_ALERT_2_G Number of HKL-OMIT Records in Embedded .res File         25 Note
                8  9  5,   9  9  7,  10  8  6,   9  9  3,  10  8  3,  11  5  1,
                9  9  2,  11  4  0,  11  6  5, -11 -4  1,   8 10  8,   8  9  0,
               -7-10  1,   9  8  5,  10  7  1,   7 10  0,  10  7  0,   9  9  8,
              -10 -7  1,   9  9  5,  -9 -8  1,   7  9  4,  11  7  9,   7  9  2,
                8  9  4,
PLAT941_ALERT_3_G Average HKL Measurement Multiplicity ..........        4.6 Low
PLAT969_ALERT_5_G The 'Henn et al.' R-Factor-gap value ..........      3.747 Note
              Predicted wR2: Based on SigI**2  4.75 or SHELX Weight 16.62
PLAT978_ALERT_2_G Number C-C Bonds with Positive Residual Density.          0 Info
PLAT994_ALERT_1_G SHELXL .ins Contains no or MERG 0 Instruction ..          ! Note
```

```
   0 ALERT level A = Most likely a serious problem - resolve or explain
   0 ALERT level B = A potentially serious problem, consider carefully
   5 ALERT level C = Check. Ensure it is not caused by an omission or oversight
  30 ALERT level G = General information/check it is not something unexpected

   1 ALERT type 1 CIF construction/syntax error, inconsistent or missing data
  12 ALERT type 2 Indicator that the structure model may be wrong or deficient
   8 ALERT type 3 Indicator that the structure quality may be low
  11 ALERT type 4 Improvement, methodology, query or suggestion
   3 ALERT type 5 Informative message, check
```

Publication of your CIF

You should attempt to resolve as many as possible of the alerts in all categories. Often the minor alerts point to easily fixed oversights, errors and omissions in your CIF or refinement strategy, so attention to these fine details can be worthwhile. In order to resolve some of the more serious problems it may be necessary to carry out additional measurements or structure refinements. However, the nature of your study may justify the reported deviations from journal submission requirements and the more serious of these should be commented upon in the discussion or experimental section of a paper or in the "special_details" fields of the CIF. *checkCIF* was carefully designed to identify outliers and unusual parameters, but every test has its limitations and alerts that are not important in a particular case may appear. Conversely, the absence of alerts does not guarantee there are no aspects of the results needing attention. It is up to the individual to critically assess their own results and, if necessary, seek expert advice.

If you wish to submit your CIF for publication in Acta Crystallographica Section C or E, you should upload your CIF via the web. If you wish to submit your CIF for publication in IUCrData you should upload your CIF via the web. If your CIF is to form part of a submission to another IUCr journal, you will be asked, either during electronic submission or by the Co-editor handling your paper, to upload your CIF via our web site.

PLATON version of 15/01/2026; check.def file version of 02/01/2026

Datablock 1 - ellipsoid plot

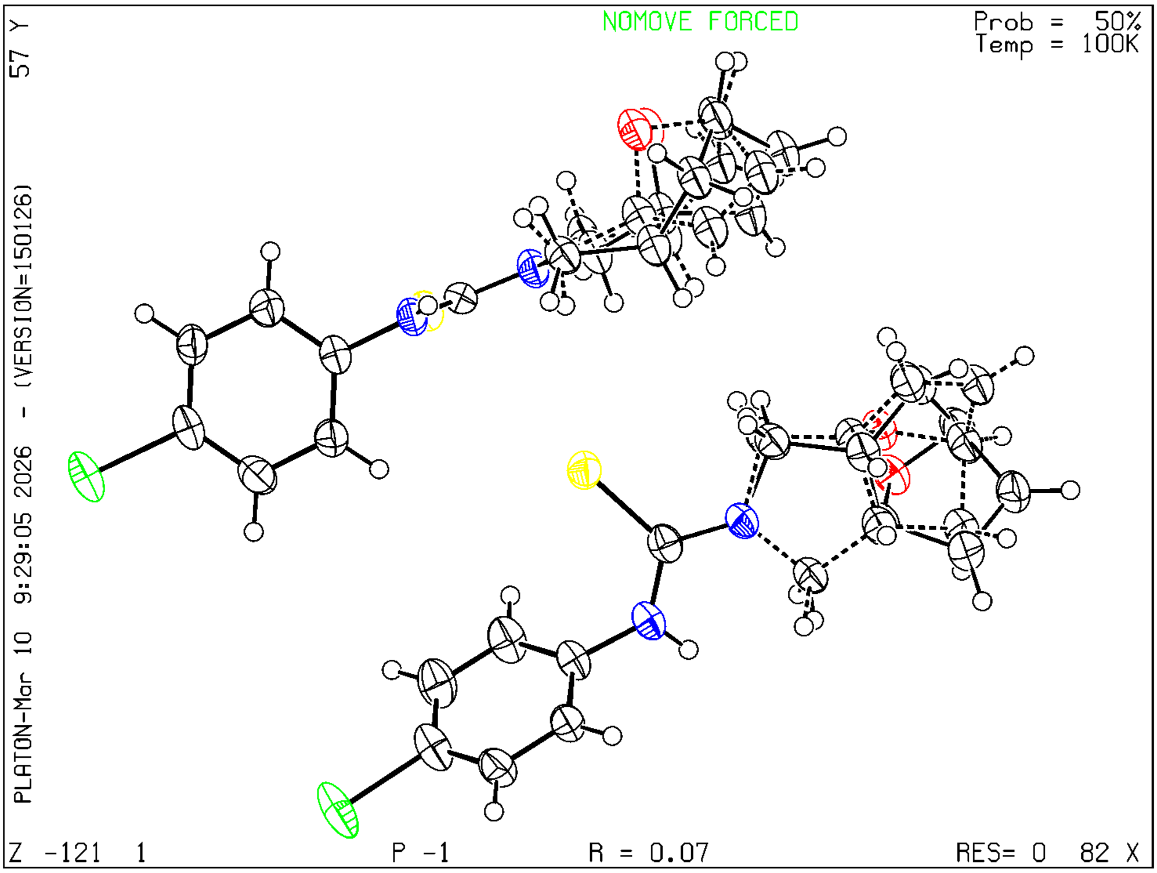

Supplement: Supplementary file 3 [file e-82-00572-sup3.pdf]
