# Supplementary material for: Synthesis and crystal structure analysis of (3aRS,6RS,7aRS)-N-(4-bromo­phen­yl)-1,6,7,7a-tetra­hydro-3a,6-ep­oxy­iso­indole-2(3H)-carboseleno­amide
Source: Acta Crystallogr E Crystallogr Commun. 2026 May 7;82(Pt 6):572–7. doi: 10.1107/S2056989026004299 (PMC13239016; doi:10.1107/S2056989026004299)
Supplement: Supplementary file 4 [file e-82-00572-sup4.pdf]

Synthesis and crystal structure analysis of (3aRS,6RS,7aRS)-*N*-(4-bromo-phenyl)-1,6,7,7a-tetrahydro-3a,6- epoxyisoindole-2(3*H*)-carboselenoamide

Dimitrii M. Shchevnikov,[a] Atash V. Gurbanov,[b] Victor N. Khrustalev,[ac] Menberu Mengesha Woldemariam,[d]* Tuncer Hökelek[e] and Roman A. Litvinov[fg]

[a]RUDN University, 6 Miklukho-Maklaya St., Moscow 117198, Russian Federation, [b]Excellence Center, Baku State University, Z. Khalilov Str. 33, AZ 1148, Azerbaijan, [c]Zelinsky Institute of Organic Chemistry of RAS, Leninsky Prospect 47, Moscow 119991, Russian Federation, [d]Department of Physics, Jimma University, Jimma, Ethiopia, [e]Hacettepe University, Department of Physics, 06800 Beytepe-Ankara, Türkiye, [f]Volgograd State Medical University, 1, Pl. Pavshikh Bortsov Square, Volgograd 400131, Russian Federation, and [g]LLC <<InnoVVita>>, Office 401, Room 2, 6 Komsomolskaya St., Volgograd 400066, Russian Federation

Correspondence email: menberu.mengesha@ju.edu.et

Abstract

The asymmetric unit of the title compound, $C_{15}H_{15}BrN_2OSe$, (**3**) contains two crystallographically independent molecules, where the cyclohexene and pyrrole rings are in boat and envelope conformations, respectively. In crystal, intermolecular C—H···O and N–H···Se hydrogen bonds link the molecules into a network, enclosing $R^2_2(20)$, $R^3_3(18)$ and $R^4_4(4)$ ring motifs. Furthermore, the C—H···π(ring) interactions help to consolidate the packing within the crystal. Hirshfeld surface analysis revealed that the most important contributions for the crystal packing are from H···H, H···C/C···H, H···Br/Br···H and H···Se/Se···H interactions.

1. Chemical context

Fibrotic diseases contribute to global mortality (Mutsaers *et al.*, 2023) and are considered as a chronic disease (Wang *et al.*, 2024). Oxidative stress is a recognized driver of fibrosis progression (Cheresh *et al.*, 2013). Scavenging reactive oxygen species (ROS) with antioxidants can intervene fibrosis (Morry *et al.*, 2017). Isoindole-based scaffolds are of interest as platforms for the development of new antioxidant therapeutics, given evidence of antioxidant activity in certain members of this class (Yakan *et al.*, 2023). Epoxidation of the isoindole core represents a promising avenue for molecular design. Accordingly, the antioxidant properties of such compounds are the subject of interest. Early studies have shown that hydrogenated isoindole-7-carboxylic acids can inhibit protein glycation (Ibragimova *et al.*, 2024), a process mechanistically linked to oxidative stress (Cho *et al.*, 2007). Recent studies show that introduction of N-substituted isoindole moiety to a chromone scaffold could produce polycyclic compounds possessing significant antibacterial properties, especially aginst Gram-negative bacteris, such as *E. coli* (Parida *et al.*, 2025). Organoselenium compounds have long been studied for their biological activities, they have shown themselves having antioxidants (Batabyal *et al.*, 2024) and anticancer (Ahn *et al.*, 2006) activities, acting as insulin analogues, cytostatic agents, uridine phospholipase inhibitors *etc*. One of the fields of synthetic organic chemistry that currently attracts the most attention is the search for synergy of bioactivity in poly-pharmacophoric compounds. Such interest drove us to seek paths of combining selenourea and isoindole moieties into a singular entity. Building on the isoindole core, subsequent elaboration led to the development of (3aRS, 6RS, 7aRS)-*N*-(4-bromophenyl)-1,6,7,7a- tetrahydro-3a,6-epoxyisoindole-2(3*H*)-carboselenoamide (**3**), a new and promising representative of the series. The attached selenium atom can participate in intermolecular chalcogen bonding in crystal packing of **3** (Gurbanov *et al.*, 2020, 2022, 2023). The selenoderivative (**3**) was prepared in one stage from commercially available allylfurfurylamine (**1**) and 1-bromo-4-isoselenocyanobenzene (**2**) (Fig. 1). The intermediate open-chain carboelenoamide underwent fast thermic intermolecular [4 + 2] cycloaddition of the allyl moiety to the furan frag-ment (the IMDAF reaction) to give the cyclic product (**3**) (Nadirova *et al.*, 2021; Zubkov *et al.*, 2009). The structure

of the target molecule was proved additionally using the NMR method, including spectra on ^{77}Se nuclei. All NMR spectra of (**3**) are complicated by amide tautomerism, which occurs in the molecule due to the difficult rotations of fragments around N—C(Se) bonds. Herein, we have reported the synthesis, molecular and crystal structures together with the Hirshfeld surface analysis of the title compound, (**3**).

2. Structural commentary

The asymmetric unit of the title compound (**3**) contains two crystallographically independent molecules (Fig. 2), where the two epoxyisoindole fragments are disordered over two sets of sites (Fig. 3). In molecules (**1**) and (**2**), the planar phenyl, (C9–C14), rings are oriented at a dihedral angle of 47.84 (5)°. Br1 atoms are −0.0688 (6) Å (in **1**) and 0.0335 (5) Å (in **2**) away from the corresponding rings planes. The six-membered nonplanar (C3a/C4—C7/C7a) rings are in boat conformations with the puckering parameters of [Q_T = 0.944 (9) Å, θ = 90.8 (5)° and φ = 359.4 (6)° (for molecule **1**)] and [Q_T = 0.941 (8) Å, θ = 90.3 (5)° and φ = 1.0 (5)° (for molecule **2**)] (Figs. 4 *a* and *b*). On the other hand, the five-membered nonplanar, [(C1/C3/C3a/C7a/N2) (Figs. 4 *c* and *d*), (O1/C3a/C4—C6) and (O1/C3a/C6/C7/C7a)], rings are in envelope conformations with the puckering parameters of [φ = 82.4 (18)° (in **1**) and φ = 88.6 (15)° (in **2**)], [φ = 2.2 (11)° (in **1**) and φ = 359.4 (9)° (in **2**)] and [φ = 180.4 (9)° (in **1**) and φ = 181.5 (8)° (in **2**)], where atoms [C7a, O1, O1] are at the flap positions and they are [−0.5008 (5) Å, 0.7747 (6) Å, −0.8521 (6) Å (in **1**)] and [−0.4787 (5) Å, −0.7659 (6) Å, −0.8543 (6) Å (in **2**) away from the best least-squares planes of the other four atoms of the corresponding rings, respectively. There are not any significant difference between the bond distances in both of the molecules **1** and **2**. But, the C8–N1–C9 [126.4 (5)° and 123.3 (4)°], C8–N2–C1 [124.7 (6)° and 123.8 (5)°], C3–N2–C1 [111.4 (5)° and 112.4 (5)°], N2–C8–N1 [117.0 (5)° and 118.0 (5)°], N1–C8–Se1 [121.9 (4)° and 120.5 (4)°], C14–C9–C10 [119.8 (5)° and 121.3 (5)°], C14–C9–N1 [118.6 (5)° and 119.3 (5)°], C10–C9–N1 [121.5 (5)° and 119.4 (5)°], C11–C10–C9 [120.0 (5)° and 119.3 (5)°] and C13–C12–C11 [121.8 (5) and 122.0 (5)°] bond angles are significantly different in both molecules **1** and **2**.

Both epoxyisoindole fragments are disordered over two sets of sites. Atoms C1, N2, C3, C3A, C4, C5, C6, C7, C7A, O1, H1A, H1B, H3A, H3B, H4, H5, H6, H7A, H7B and H7AA were disordered over two positions in both molecules **1** and **2** of the asymmetric unit, and they were refined with the occupancy ratios of 0.725 (7):0.275 (7) and 0.831 (6):0.169 (6), respectively. Refinement of this disorder resulted in a meaningful model lowering the previous large difference electron density from 1.685 e.Å^{-3} to 1.381 e.Å^{-3}. On the other hand, the large residuals are now limited to the area around Se atoms, and the *R* value was converged to 0.0699 instead of 0.0741. For a more compherensible and visual comparison of the two molecules present in the asymmetric unit, an overlay plot is given in Fig. 5. The differences between the two molecules are clearly seen in the conformations about the carboselenoamide moieties, with the torsion angles of C9–N1–C8–N2 [171.6 (5)° and 175.2 (5)°], C3–N2–C8–N1 [3.4 (9)° and 174.4 (6)°], C1–N2–C8–N1 [−173.5 (6)° and 0.1 (10)°], C10–C9–N1–C8 [67.9 (8)° and −98.1 (6)°] and C14–C9–N1–C8 [−115.1 (6)° and 83.8 (7)°] for molecules **1** and **2**, respectively, so that non of the rings overlap exactly.

Due to the poor solubility of the title compound (**3**), elevated temperatures were required to record the NMR spectra. The sample was heated to ensure complete dissolution (Fig. 6).

3. Supramolecular features

In the crystal, intermolecular C—H···O and N—H···Se hydrogen bonds (Table 2) link the molecules into a network, enclosing $R^2_2(20)$, $R^3_3(18)$ and $R^4_4(4)$ ring motifs (Etter *et al.*, 1990) (Fig. 7). Further the C—H···π(ring) interactions (Table 2) help to consolidate the packing within the crystal. On the other hand, there are intermolecular Br···Br halogen bonds [3.587 Å and 3.632 Å], in which they are slightly lower than the sum of van der Waals radii of the Br atoms [3.70

Å] leading to a supramolecular tetramer (Fig. 8). Due to the weak character of the Br···Br interactions, the C–Br···Br angles [132.9 (2)° and 151.3 (2)°] are far from 180°, the directionality term of the halogen bonding.

4. Hirshfeld surface analysis

For visualizing the intermolecular interactions in the crystal of title compound (**3**), Hirshfeld surface (HS) analyses were carried out by using Crystal Explorer 17.5 (Spackman *et al.*, 2021). It is noted that only the major components of the disordered parts of the epoxyisoindole fragments were taken into account for the analysis. In the HSs plotted over d_{norm} (Figs. 9 *a* and *b*), the contact distances equal, shorter and longer with respect to the sum of van der Waals radii are shown by the white, red and blue colours, respectively. The present red spots indicate their roles as the respective donors and/or acceptors in hydrogen bonding, as discussed. In addition shape index was used to identify possible $\pi\cdots\pi$ stacking and C—H···π(ring) interactions as 'red π-holes' which are related to the electron ring interactions between the C—H groups with the centroid of the aromatic rings of the neighboring molecules. Fig. 10 clearly suggests that there are C—H···π(ring) interactions in the title compound but no $\pi\cdots\pi$ interactions. The overall two-dimensional fingerprint plots are shown in Figs. 11*a* and 12*a* and those delineated into H ··· H, H ··· C/C ··· H, H ··· Br/Br ··· H, H ··· Se/Se ··· H, H ··· O/O ··· H, C ··· C, H ··· N/N ··· H, Br ··· Br, O ··· O, C ··· O/O ··· C, C ··· Se/Se ··· C, N ··· Se/Se ··· N, Se ··· Se and O ··· Br/Br ··· O interactions (for molecule **1**) and H ··· H, H ··· C/C ··· H, H ··· Se/Se ··· H, H ··· Br/Br ··· H, H ··· O/O ··· H, C ··· C, Br ··· Br, H ··· N/N ··· H, O ··· O, C ··· O/O ··· C, N ··· Se/Se ··· N and C ··· Se/Se ··· C (for molecule **2**) interactions are illustrated in Figs. 11 (*b*–*l*) and Figs. 12 (*b*–*m*) for molecules **1** and **2**, respectively. Their contributions to the HSs are presented in Table 3. Comparison of their percentages for molecules **1** and **2** shows that there are no significant differences due to the similar values of the close contacts.

5. Synthesis and crystallization

Starting *N*-(furan-2-ylmethyl)prop-2-en-1-amine (**1**) (100 mg, 0.7 mmol) was dissolved in benzene (5 ml) at r.t. 1-bromo-4-isoselenocyanatobenzene (**2**) (190 mg, 0.7 mmol) was added to the solution, the reaction was refluxed for 6 h (TLC control). The resulting mixture ws cooled, and formation of solid was observed. The crystals were filtered off, washed with diethyl ether (3 × 5 ml), dried under vacuum and then at the air. The target product (**3**) did not require further purification, yield 44%, 122.9 mg (0.321 mmol), colourless crystals, m.p. 490–491 K. A single-crystal of the title compound was grown from a mixture of EtOH/DMF. IR (KBr), ν (cm^{-1}): 3142, 1530, ^{1}H NMR (300.1 MHz, DMSO-d_6, 373 K) (J, Hz): δ 8.99 (br.s, 1H), 7.50–7.38 (m, 4H), 6.51 (d, J = 5.7 Hz, 1H), 6.46 (dd, J = 5.7, 1.7 Hz, 1H), 5.08 (dd, J = 4.4, 1.7 Hz, 1H), 4.38–4.16 (m, 3H), 3.25 (br.dd, J = 11.4, 9.7 Hz, 1H), 2.33–2.23 (m, 1H), 1.79 (ddd, J = 11.7, 4.4, 2.8 Hz, 1H), 1.50 (dd, J = 11.7, 7.5 Hz, 1H) p.p.m.. $^{13}C\{^{1}H\}$ NMR (75.5 MHz, DMSO-d_6, 373 K): δ 178.2, 141.3, 137.8, 134.3, 131.1 (2 C), 128.9 (2 C), 122.0, 94.1, 80.2, 57.3, 54.0, 41.5, 32.3. $^{77}Se\{^{1}H\}$ NMR (57.2 MHz, DMSO-d_6, 373 K): δ 303.0. ^{1}H NMR (700.2 MHz, DMSO-d_6, 353 K): (J, Hz): δ 9.12 (br.s, 1H), 7.49 (d, J = 8.1 Hz, 2H), 7.38 (d, J = 8.1 Hz, 2H), 6.52 (br.d, J = 5.2 Hz, 1H), 6.48 (br.d, J = 5.2 Hz, 1H), 5.06 (br.d, J = 2.9 Hz, 1H), broaden H-1,3 signals ~ 4.50–4.00 (m, 3H), 3.23 (br.s, 1H), 2.28 (br.s, 1H), 1.78 (br.d, J = 10.5 Hz, 1H), 1.49 (br.d, J = 11.0, 7.6 Hz, 1H) p.p.m.. ^{13}C NMR (176.1 MHz, DMSO-d_6, 353 K): δ signals of 4 carbon atoms of the epoxyisoindole moiety are very broad and absent in the spectra, 177.8, 141.3, 137.8, 134.3, 131.2 (2 C), 129.0 (2 C), 117.9 80.2, 32.3. $^{77}Se\{^{1}H\}$ NMR (57.2 MHz, DMSO-d_6): δ the signal of the Se nuclei are duplicated due to amide rotamerism 286.0, 283.5 p.p.m.. MS (ESI) m/*z*: $[M]^+$ 399 $[M\text{+H}]^+$.

6. Refinement

Crystal data, data collection and structure refinement details are summarized in Table 1. The N– and C-bond hydrogen atom positions were calculated geometrically at distances of 0.88 (for NH), 1.00 (for methine CH), 0.95 (for aromatic

CH) and 0.99 (for methylene CH) and refined using a riding model by applying the constraint of U_{iso} = 1.2 × U_{eq} (C, N). However, the disorder was trace and due to the presence of a superposition of the two different conformers, namely, the conformer **1** and the inverted conformer **2** (see Figure 4). It was impossible to split their positions within the frame of the experiment performed.

Table 1

Experimental details

Crystal data	
Chemical formula	$C_{15}H_{15}BrN_2OSe$
M_r	398.15
Crystal system, space group	Triclinic, $P\overline{1}$
Temperature (K)	100
a, *b*, *c* (Å)	9.7367 (4), 10.3981 (4), 15.7685 (5)
α, β, γ (°)	73.059 (3), 76.870 (3), 84.140 (4)
V (Å^3)	1486.15 (10)
Z	4
Radiation type	Cu *K*α
μ (mm^{-1})	6.54
Crystal size (mm)	0.30 × 0.06 × 0.03
Data collection	
Diffractometer	Rigaku XtaLAB Synergy-S, HyPix-6000HE area-detector
Absorption correction	Gaussian<br>*CrysAlis PRO* 1.171.44.118a (Rigaku Oxford Diffraction, 2025). Numerical absorption correction based on gaussian integration over a multifaceted crystal model.
T_{min}, T_{max}	0.353, 1.000
No. of measured, independent and observed [$I > 2\sigma(I)$] reflections	28318, 6210, 5303
R_{int}	0.101
$(\sin \theta/\lambda)_{max}$ (Å^{-1})	0.639
Refinement	
$R[F^2 > 2\sigma(F^2)]$, $wR(F^2)$, S	0.070, 0.178, 1.07
No. of reflections	6210
No. of parameters	525
No. of restraints	930
H-atom treatment	H-atom parameters constrained
$\Delta\rho_{max}$, $\Delta\rho_{min}$ (e Å^{-3})	1.38, −1.22

Computer programs: *CrysAlis PRO* 1.171.41.117a (Rigaku OD, 2021), *SHELXT* (Sheldrick, 2015), *SHELXL* (Sheldrick, 2015), *SHELXTL* (Sheldrick, 2015).

Table 2

Hydrogen-bond geometry (Å, º)

Cg8 and Cg17 are the centroids of the C9_1···C14_1 and C9_2···C14_2 rings.

D—H···*A*	*D*—H	H···*A*	*D*···*A*	*D*—H···*A*

N1_1—H1_1···Se1_2^{i}	0.88	2.67	3.460 (5)	151
C11_1—H11_1···O1_1^{i}	0.95	2.34	3.283 (8)	174
C11_1—H11_1···O1*B*_1^{i}	0.95	2.25	3.176 (14)	165
N1_2—H1_2···Se1_1^{ii}	0.88	2.64	3.393 (5)	144
C11_2—H11_2···O1_2^{iii}	0.95	2.44	3.368 (7)	167
C11_2—H11_2···O1*B*_2^{iii}	0.95	2.48	3.36 (2)	154
C1_1—H1*A*_1···*Cg*17^{ii}	0.99	2.83	3.720 (11)	150
C3_2—H3*B*_2···*Cg*8^{i}	0.99	2.81	3.724 (9)	154

Symmetry codes: (i) −*x*, −*y*+1, −*z*+1; (ii) −*x*+1, −*y*+1, −*z*+1; (iii) −*x*+1, −*y*+2, −*z*+1.

Table 3

Comparison of the percentages for molecules **1** and **2**.

Contacts	**1**	**2**
H···H	51.5	47.6
H···C/C···H	14.1	17.0
H···Br/Br···H	10.5	9.6
H···Se/Se···H	10.1	9.9
H···O/O···H	6.7	6.1
C···C	2.4	3.8
H···N/N···H	1.1	1.3
Br.·Br	1.0	2.4
O···O	1.0	0.9
C···O/O···C	0.5	0.5
C···Se/Se···C	0.4	0.4
N···Se/Se···N	0.3	0.4
Se···Se	0.2	0.0
O···Br/Br···O	0.1	0.0

Acknowledgements

This work was provided by the Russian Science Foundation and the Administration of Volgograd oblast (Project No. 24–24-201112, https://rscf.ru/project/24–24-201112/), as well as the Baku State University. T. H. is also grateful to Hacettepe University Scientific Research Project Unit (Grant No. 013 D04 602 004). The author's contributions are as follows. Conceptualization, AVG and MMW; synthesis, DMS and RAL; X-ray analysis, AVG, VKN and TH; Hirshfeld surface analysis, TH; writing (review and editing of the manuscript) AVG, DMS, RAL and TH, supervision, AVG, TH and MMW.

Funding information

References

Ahn, H. J., Koketsu, M., Yang, E. M., Kim, Y. M., Ishihara, H. & Yang, H. O. (2006). *J. Cell Biochem.* **99**, 807–815.

Batabyal, M., Chaurasia, D., Panda, P. R., Jha, R. K., Kadu, R. & Kumar, S. (2024). *J. Org. Chem.* **89**, 14328–14340.

Cheresh, P., Kim, S. J., Tulasiram, S. & Kamp, D. W. (2013). *Biochimica et Biophysica (BBA)-Molecular Basis of Disease* **1832**, 1028–1040.

Cho, S. J., Roman, G., Yeboah, F. & Konishi, Y. (2007). *Curr. Med. Chem.* **14**, 1653–1671.

Etter, M. C., MacDonald, J. C. & Bernstein, J. (1990). *Acta Cryst.* E**46**, 256–262.

Gurbanov, A. V., Aliyeva, V. A., Gomila, R. M., Frontera, A., Mahmudov, K. T. & Pombeiro, A. J. (2023). *Cryst. Growth Des.* **23**, 7335–7344.

Gurbanov, V. A., Kuznetsov, M. L., Mahmudov, K. T., Pombeiro, A. J. L. & Resnati, G. (2020). *Chem. Eur. J.* **26**, 14833–14837.

Gurbanov, V. A., Kuznetsov, M. L., Resnati, G., Mahmudov, K. T. & Pombeiro, A. J. L. (2022). *Cryst. Growth Des.* **22**, 3932–3940.

Ibragimova, U., Valuisky, N., Sorokina, S., Zhukova, X., Railberg, V. & Litvinov, R. (2024). *Molecular Biology* **58**, 1157–1164.

Morry, J., Ngamcherdtrakul, W. & Yantasee, W. (2017). *Redox Biology* **11**, 240–253.

Mutsaers, H. A. M., Merrid, C., Norregaard, R. & Plana- Ripoll, O. (2023). *J. Transl. Med.* **21**, 818.

Nadirova, M. A., Khanova, A. V., Zubkov, F. I., Mertsalov, D. F., Kolesnik, I. A., Petrkevich, S. K., Potkin, V. I., Shetnev, A. A., Prsnukhina, S. I., Sinelshchikova, A. A., Grigoriev, M. S. & Zaytsev, V. P. (2021). *Tetrahedron* **85**, 132032–132049.

Parida, S. P., Mohapatra, S., Mohapatra, S., Behera, T., Nayak, S. & Sahoo, C. R. (2025). *RSC Adv.* **15**, 14499–14517.

Rigaku OD (2021). *CrysAlis PRO 1.171.44.118a* and *CrysAlis PRO 1.171.41.117a*.

Sheldrick, G. M. (2015*a*). *Acta Cryst.* A**71**, 3–8.

Sheldrick, G. M. (2015*b*). *Acta Cryst.* C**71**, 3–8.

Spackman, P. R., Turner, M. J., McKinnon, J. J., Wolff, S. K., Grimwood, D. J., Jayatilaka, D. & Spackman, M. A. (2021). *J. Appl. Cryst.* **54**, 1006–1011.

Wang, J., Li, K., Hao, D., Li, x., Zhu, Y., Yu, H. & Chen, H. (2024). *MedComm* **5**, e744.

Yakan, H., Ozturk, S., Tolgay, E. U., Yenigun, S., Marah, S., Doruk, T., Ozen, T. & Kutuk, H. (2023). *Acta Chim. Slov.* **70**, 29–43.

Zubkov, F. I., Ershova, J. D., Orlova, A. A., Zaytsev, V. P., Nikitina, E. V., Peregudov, A. S., Gurbanov, A. V., Borisov, R. S., Khrut-stalev, V. N., Maharramov, A. M. & Varlamov, A. V. (2009). *Tetrahedron* **65**, 3739–3803.

Figure 1

Reaction scheme for obtaining the title compound, (**3**).

Figure 2

The asymmetric unit of the title compound (**3**) with atom-numbering scheme and 50% probability ellipsoids.

Figure 3

The molecular diagram drawn only for molecule **1** in the asymmetric unit showing the disordering in the epoxyisoindole fragment over two sets of sites.

Figure 4

Conformations of (*a*) cyclohexene (in **1**), (*b*) cyclohexene (in **2**), (*c*) pyrrole (in **1**) and (*d*) pyrrole (in **2**) rings.

Figure 5

An overlay plot of the two molecules (**1** and **2**) present in the asymmetric unit.

Figure 6

The NMR spectra recorded at elevated temperatures due to the poor solubility of the title compound (**3**).

Figure 7

The partial packing diagram of the title compound (**3**). Intermolecular C—H···O and N—H···Se hydrogen bonds are shown as dashed lines. Nonbonding H atoms have been omitted for clarity.

Figure 8

The intermolecular Br···Br halogen bonds leading to a supramolecular tetramer.

Figure 9

Views of the three-dimensional Hirshfeld surfaces for molecules (*a*) **1** and (*b*) **2** plotted over d_{norm} in the ranges from −1.8335 to 1.4177 a.u. and −1.8518 to 1.1855 a.u., respectively.

Figure 10

Hirshfeld surfaces for molecules **1** and **2** plotted over shape-index for two orientations showing the C—H···π(ring) interactions.

Figure 11

The full two-dimensional fingerprint plots for molecule **1**, showing (*a*) all interactions, and delineated into (*b*) H ··· H, (*c*) H ··· C/C ··· H, (*d*) H ··· Br/Br ··· H, (*e*) H ··· Se/Se ··· H, (*f*) H ··· O/O ··· H, (*g*) C ··· C, (*h*) H ··· N/N ··· H, (i) Br ··· Br, (*j*) O ··· O, (*k*) C ··· O/O ··· C, (*l*) C ··· Se/Se ··· C, (*m*) N ··· Se/Se ··· N, (*n*) Se ··· Se and (*o*) O ··· Br/Br ··· O interactions. The d_i and d_e values are the closest internal and external distances (in Å) from given points on the Hirshfeld surface contacts.

Figure 12

The full two-dimensional fingerprint plots for molecule **2**, showing (*a*) all interactions, and delineated into (*b*) H ··· H, (*c*) H ··· C/C ··· H, (*d*) H ··· Se/Se ··· H, (*e*) H ··· Br/Br ··· H, (*f*) H ··· O/O ··· H, (*g*) C ··· C, (*h*) Br ··· Br, (i) H ··· N/N ··· H, (*j*) O ··· O, (*k*) C ··· O/O ··· C, (*l*) N ··· Se/Se ··· N and (*m*) C ··· Se/Se ··· C interactions. The d_i and d_e values are the closest internal and external distances (in Å) from given points on the Hirshfeld surface contacts.

supporting information

Synthesis and crystal structure analysis of (3aRS,6RS,7aRS)-*N*-(4-bromo-phenyl)-1,6,7,7a-tetrahydro-3a,6- epoxyisoindole-2(3*H*)-carboselenoamide

Computing details

Data collection: *CrysAlis PRO* 1.171.41.117a (Rigaku OD, 2021); cell refinement: *CrysAlis PRO* 1.171.41.117a (Rigaku OD, 2021); data reduction: *CrysAlis PRO* 1.171.41.117a (Rigaku OD, 2021); program(s) used to solve structure: *SHELXT* (Sheldrick, 2015); program(s) used to refine structure: *SHELXL* (Sheldrick, 2015); molecular graphics: *SHELXTL* (Sheldrick, 2015); software used to prepare material for publication: *SHELXTL* (Sheldrick, 2015).

(1)

Crystal data

$C_{15}H_{15}BrN_2OSe$
M_r = 398.15
Triclinic, $P\bar{1}$
a = 9.7367 (4) Å
b = 10.3981 (4) Å
c = 15.7685 (5) Å
α = 73.059 (3)°
β = 76.870 (3)°
γ = 84.140 (4)°
V = 1486.15 (10) Å^3
Z = 4
F(000) = 784
D_x = 1.780 Mg m^{-3}
Cu $K\alpha$ radiation, λ = 1.54184 Å
Cell parameters from 14508 reflections
θ = 3.0–79.4°
μ = 6.54 mm^{-1}
T = 100 K
Prismatic needle, colourless
0.30 × 0.06 × 0.03 mm

Data collection

Rigaku XtaLAB Synergy-S, HyPix-6000HE area-detector diffractometer
Radiation source: micro-focus sealed X-ray tube
φ and ω scans
Absorption correction: gaussian *CrysAlis PRO* 1.171.44.118a (Rigaku Oxford Diffraction, 2025). Numerical absorption correction based on gaussian integration over a multifaceted crystal model.
T_{min} = 0.353, T_{max} = 1.000
28318 measured reflections
6210 independent reflections
5303 reflections with $I > 2\sigma(I)$
R_{int} = 0.101
θ_{max} = 80.4°, θ_{min} = 3.0°
h = −12→12
k = −13→12
l = −20→20

Refinement

Refinement on F^2
Least-squares matrix: full
$R[F^2 > 2\sigma(F^2)]$ = 0.070
$wR(F^2)$ = 0.178
S = 1.07
6210 reflections
525 parameters
930 restraints
Primary atom site location: difference Fourier map
Secondary atom site location: difference Fourier map
Hydrogen site location: inferred from neighbouring sites
H-atom parameters constrained
$w = 1/[\sigma^2(F_o^2) + (0.0974P)^2 + 4.6292P]$ where $P = (F_o^2 + 2F_c^2)/3$
$(\Delta/\sigma)_{max}$ = 0.001
$\Delta\rho_{max}$ = 1.38 e Å^{-3}
$\Delta\rho_{min}$ = −1.22 e Å^{-3}

Special details

Geometry. All e.s.d.'s (except the e.s.d. in the dihedral angle between two l.s. planes) are estimated using the full covariance matrix. The cell e.s.d.'s are taken into account individually in the estimation of e.s.d.'s in distances, angles and torsion angles; correlations between e.s.d.'s in cell parameters are only used when they are defined by crystal symmetry. An approximate (isotropic) treatment of cell e.s.d.'s is used for estimating e.s.d.'s involving l.s. planes.

Fractional atomic coordinates and isotropic or equivalent isotropic displacement parameters (Å²)

	x	*y*	*z*	U_{iso}*/U_{eq}	Occ. (<1)
Br1_1	−0.00774 (8)	0.15550 (10)	0.91076 (4)	0.0679 (3)	
Se1_1	0.30048 (7)	0.46618 (6)	0.46015 (4)	0.04111 (18)	
N1_1	0.0925 (5)	0.2751 (5)	0.5014 (3)	0.0388 (9)	
H1_1	0.033003	0.235262	0.483781	0.047*	
C1_1	0.3049 (11)	0.4122 (9)	0.2719 (6)	0.0392 (18)	0.725 (7)
H1A_1	0.399434	0.399056	0.287253	0.047*	0.725 (7)
H1B_1	0.282548	0.509933	0.251227	0.047*	0.725 (7)
N2_1	0.1970 (5)	0.3465 (4)	0.3511 (3)	0.0366 (8)	0.725 (7)
C3_1	0.1025 (12)	0.2681 (12)	0.3260 (7)	0.039 (2)	0.725 (7)
H3A_1	0.002571	0.296332	0.344365	0.046*	0.725 (7)
H3B_1	0.114863	0.170718	0.355145	0.046*	0.725 (7)
C3A_1	0.1457 (8)	0.2983 (8)	0.2244 (5)	0.0414 (14)	0.725 (7)
C4_1	0.1306 (11)	0.2055 (9)	0.1700 (6)	0.0455 (17)	0.725 (7)
H4_1	0.116417	0.111466	0.191433	0.055*	0.725 (7)
C5_1	0.1415 (9)	0.2833 (8)	0.0854 (5)	0.0469 (16)	0.725 (7)
H5_1	0.139614	0.255589	0.033326	0.056*	0.725 (7)
C6_1	0.1574 (10)	0.4242 (9)	0.0887 (5)	0.0462 (17)	0.725 (7)
H6_1	0.127456	0.497280	0.038396	0.055*	0.725 (7)
C7_1	0.3096 (9)	0.4342 (9)	0.1010 (5)	0.0459 (15)	0.725 (7)
H7A_1	0.381260	0.398371	0.057215	0.055*	0.725 (7)
H7B_1	0.330864	0.527874	0.095136	0.055*	0.725 (7)
C7A_1	0.3005 (8)	0.3443 (8)	0.1988 (5)	0.0413 (14)	0.725 (7)
H7AA_1	0.369423	0.265808	0.200985	0.050*	0.725 (7)
O1_1	0.0777 (6)	0.4217 (6)	0.1781 (3)	0.0421 (12)	0.725 (7)
C1B_1	0.120 (3)	0.253 (3)	0.3256 (16)	0.039 (3)	0.275 (7)
H1C_1	0.022337	0.287147	0.321875	0.047*	0.275 (7)
H1D_1	0.118223	0.162414	0.368816	0.047*	0.275 (7)
N2B_1	0.1970 (5)	0.3465 (4)	0.3511 (3)	0.0366 (8)	0.275 (7)
C3B_1	0.291 (3)	0.436 (3)	0.2759 (12)	0.040 (3)	0.275 (7)
H3C_1	0.390635	0.419194	0.281893	0.048*	0.275 (7)
H3D_1	0.263991	0.532041	0.271727	0.048*	0.275 (7)
C3AB_1	0.2664 (17)	0.3980 (17)	0.1958 (9)	0.041 (2)	0.275 (7)
C4B_1	0.3772 (18)	0.404 (2)	0.1118 (10)	0.044 (3)	0.275 (7)
H4B_1	0.476242	0.407838	0.105237	0.053*	0.275 (7)
C5B_1	0.3097 (18)	0.401 (2)	0.0490 (10)	0.044 (3)	0.275 (7)
H5B_1	0.349877	0.404278	−0.012295	0.053*	0.275 (7)
C6B_1	0.155 (2)	0.393 (2)	0.0940 (11)	0.044 (2)	0.275 (7)
H6B_1	0.087964	0.426091	0.051986	0.053*	0.275 (7)
C7B_1	0.131 (3)	0.2492 (19)	0.1579 (13)	0.045 (3)	0.275 (7)
H7C_1	0.173925	0.180053	0.126932	0.054*	0.275 (7)
H7D_1	0.029384	0.232814	0.182530	0.054*	0.275 (7)
C7AB_1	0.209 (2)	0.2531 (16)	0.2329 (10)	0.042 (2)	0.275 (7)
H7AB_1	0.287638	0.183335	0.237955	0.051*	0.275 (7)

O1B_1	0.1482 (16)	0.4691 (14)	0.1595 (9)	0.042 (2)	0.275 (7)
C8_1	0.1889 (5)	0.3519 (5)	0.4356 (3)	0.0356 (10)	
C9_1	0.0777 (6)	0.2523 (6)	0.5963 (3)	0.0377 (11)	
C10_1	0.0327 (7)	0.3551 (6)	0.6380 (4)	0.0471 (13)	
H10_1	0.018502	0.444400	0.602203	0.056*	
C11_1	0.0088 (7)	0.3272 (7)	0.7308 (4)	0.0526 (15)	
H11_1	−0.022626	0.396542	0.759604	0.063*	
C12_1	0.0311 (7)	0.1963 (7)	0.7818 (4)	0.0505 (14)	
C13_1	0.0777 (7)	0.0944 (6)	0.7421 (4)	0.0443 (12)	
H13_1	0.092602	0.005481	0.778176	0.053*	
C14_1	0.1025 (6)	0.1231 (6)	0.6486 (4)	0.0389 (11)	
H14_1	0.136740	0.053811	0.620145	0.047*	
Br1_2	0.25531 (8)	0.75885 (8)	0.90577 (4)	0.0552 (2)	
Se1_2	0.18559 (6)	0.92147 (6)	0.47837 (4)	0.03988 (18)	
N1_2	0.4327 (5)	0.7680 (5)	0.5088 (3)	0.0365 (9)	
H1_2	0.515206	0.730673	0.490691	0.044*	
C1_2	0.5323 (10)	0.7390 (13)	0.3349 (5)	0.0397 (13)	0.831 (6)
H1A_2	0.543596	0.652868	0.381026	0.048*	0.831 (6)
H1B_2	0.614456	0.794349	0.325014	0.048*	0.831 (6)
N2_2	0.3984 (5)	0.8117 (5)	0.3640 (3)	0.0376 (9)	0.831 (6)
C3_2	0.3156 (9)	0.8578 (8)	0.2923 (5)	0.0425 (17)	0.831 (6)
H3A_2	0.288142	0.954533	0.282186	0.051*	0.831 (6)
H3B_2	0.229513	0.805507	0.307811	0.051*	0.831 (6)
C3A_2	0.4165 (7)	0.8321 (8)	0.2102 (4)	0.0419 (13)	0.831 (6)
C4_2	0.3733 (8)	0.8112 (8)	0.1289 (4)	0.0475 (15)	0.831 (6)
H4_2	0.283605	0.785900	0.126428	0.057*	0.831 (6)
C5_2	0.4851 (8)	0.8350 (8)	0.0626 (4)	0.0475 (14)	0.831 (6)
H5_2	0.492770	0.831504	0.002160	0.057*	0.831 (6)
C6_2	0.5986 (9)	0.8691 (8)	0.1025 (5)	0.0477 (15)	0.831 (6)
H6_2	0.676085	0.922685	0.057128	0.057*	0.831 (6)
C7_2	0.6492 (8)	0.7391 (8)	0.1676 (4)	0.0450 (14)	0.831 (6)
H7A_2	0.669275	0.663923	0.139352	0.054*	0.831 (6)
H7B_2	0.733960	0.753993	0.187783	0.054*	0.831 (6)
C7A_2	0.5188 (7)	0.7141 (8)	0.2461 (4)	0.0425 (13)	0.831 (6)
H7AA_2	0.478651	0.624617	0.256699	0.051*	0.831 (6)
O1_2	0.5165 (6)	0.9373 (5)	0.1664 (3)	0.0463 (11)	0.831 (6)
C1B_2	0.326 (4)	0.889 (4)	0.2913 (17)	0.039 (3)	0.169 (6)
H1C_2	0.354724	0.984011	0.268051	0.047*	0.169 (6)
H1D_2	0.222209	0.886858	0.311360	0.047*	0.169 (6)
N2B_2	0.3984 (5)	0.8117 (5)	0.3640 (3)	0.0376 (9)	0.169 (6)
C3B_2	0.531 (4)	0.742 (5)	0.3375 (16)	0.039 (3)	0.169 (6)
H3C_2	0.529778	0.644714	0.369489	0.047*	0.169 (6)
H3D_2	0.612221	0.781893	0.347498	0.047*	0.169 (6)
C3AB_2	0.533 (2)	0.768 (2)	0.2383 (12)	0.043 (2)	0.169 (6)
C4B_2	0.598 (3)	0.668 (2)	0.1876 (15)	0.045 (3)	0.169 (6)
H4B_2	0.625006	0.576213	0.211977	0.054*	0.169 (6)
C5B_2	0.610 (3)	0.735 (3)	0.1022 (15)	0.047 (3)	0.169 (6)
H5B_2	0.635581	0.698389	0.051758	0.056*	0.169 (6)
C6B_2	0.575 (3)	0.881 (3)	0.0988 (14)	0.046 (3)	0.169 (6)
H6B_2	0.623663	0.947474	0.043065	0.055*	0.169 (6)
C7B_2	0.412 (3)	0.899 (3)	0.1211 (14)	0.047 (3)	0.169 (6)
H7C_2	0.367144	0.864103	0.082050	0.056*	0.169 (6)

H7D_2	0.380590	0.994072	0.115919	0.056*	0.169 (6)
C7AB_2	0.381 (2)	0.811 (3)	0.2209 (14)	0.044 (3)	0.169 (6)
H7AB_2	0.322772	0.732251	0.230271	0.053*	0.169 (6)
O1B_2	0.604 (2)	0.889 (2)	0.1832 (13)	0.045 (2)	0.169 (6)
C8_2	0.3529 (6)	0.8241 (5)	0.4474 (3)	0.0345 (10)	
C9_2	0.3902 (6)	0.7659 (5)	0.6024 (3)	0.0364 (11)	
C10_2	0.4397 (6)	0.8613 (6)	0.6321 (4)	0.0419 (12)	
H10_2	0.500901	0.927874	0.590903	0.050*	
C11_2	0.3989 (6)	0.8591 (6)	0.7232 (4)	0.0412 (12)	
H11_2	0.430534	0.924927	0.744619	0.049*	
C12_2	0.3117 (6)	0.7595 (6)	0.7819 (3)	0.0396 (12)	
C13_2	0.2630 (6)	0.6619 (7)	0.7527 (4)	0.0460 (13)	
H13_2	0.203282	0.594257	0.794113	0.055*	
C14_2	0.3035 (6)	0.6655 (6)	0.6620 (4)	0.0407 (11)	
H14_2	0.272202	0.599520	0.640586	0.049*	

Atomic displacement parameters (Å^2)

	U^{11}	U^{22}	U^{33}	U^{12}	U^{13}	U^{23}
Br1_1	0.0604 (4)	0.1135 (7)	0.0255 (3)	0.0060 (4)	−0.0040 (3)	−0.0196 (3)
Se1_1	0.0526 (4)	0.0383 (3)	0.0350 (3)	−0.0026 (2)	−0.0133 (2)	−0.0107 (2)
N1_1	0.045 (2)	0.045 (2)	0.026 (2)	−0.0009 (19)	−0.0091 (18)	−0.0071 (18)
C1_1	0.046 (3)	0.039 (4)	0.031 (3)	−0.002 (3)	−0.007 (3)	−0.008 (3)
N2_1	0.046 (2)	0.0370 (19)	0.0261 (17)	−0.0026 (16)	−0.0105 (16)	−0.0048 (15)
C3_1	0.045 (4)	0.042 (4)	0.027 (3)	−0.005 (3)	−0.009 (3)	−0.004 (3)
C3A_1	0.047 (3)	0.046 (3)	0.030 (2)	−0.002 (3)	−0.010 (2)	−0.007 (2)
C4_1	0.050 (3)	0.051 (4)	0.037 (3)	−0.007 (3)	−0.007 (3)	−0.014 (3)
C5_1	0.052 (3)	0.059 (4)	0.034 (3)	−0.008 (3)	−0.013 (3)	−0.014 (3)
C6_1	0.056 (3)	0.052 (3)	0.028 (3)	−0.003 (3)	−0.009 (3)	−0.005 (3)
C7_1	0.053 (3)	0.052 (3)	0.033 (3)	−0.008 (3)	−0.008 (3)	−0.011 (3)
C7A_1	0.046 (3)	0.046 (3)	0.033 (3)	−0.003 (3)	−0.009 (2)	−0.012 (2)
O1_1	0.047 (3)	0.047 (3)	0.031 (2)	0.002 (2)	−0.011 (2)	−0.006 (2)
C1B_1	0.046 (5)	0.041 (5)	0.027 (4)	−0.003 (4)	−0.007 (4)	−0.006 (4)
N2B_1	0.046 (2)	0.0370 (19)	0.0261 (17)	−0.0026 (16)	−0.0105 (16)	−0.0048 (15)
C3B_1	0.046 (5)	0.042 (5)	0.029 (4)	0.000 (4)	−0.008 (4)	−0.008 (4)
C3AB_1	0.049 (4)	0.044 (4)	0.031 (3)	−0.002 (4)	−0.010 (3)	−0.009 (3)
C4B_1	0.053 (5)	0.047 (5)	0.031 (5)	0.000 (5)	−0.008 (5)	−0.012 (4)
C5B_1	0.054 (5)	0.050 (5)	0.029 (5)	−0.003 (5)	−0.010 (5)	−0.010 (4)
C6B_1	0.051 (4)	0.051 (4)	0.030 (3)	−0.002 (4)	−0.010 (3)	−0.009 (3)
C7B_1	0.053 (4)	0.049 (4)	0.033 (4)	−0.003 (4)	−0.011 (4)	−0.010 (4)
C7AB_1	0.049 (4)	0.046 (4)	0.031 (4)	−0.002 (4)	−0.008 (4)	−0.010 (4)
O1B_1	0.050 (4)	0.045 (4)	0.031 (3)	0.002 (4)	−0.011 (3)	−0.010 (3)
C8_1	0.038 (2)	0.036 (2)	0.031 (2)	0.008 (2)	−0.013 (2)	−0.0065 (19)
C9_1	0.039 (3)	0.046 (3)	0.027 (2)	0.004 (2)	−0.008 (2)	−0.009 (2)
C10_1	0.054 (3)	0.046 (3)	0.040 (3)	0.018 (3)	−0.012 (2)	−0.015 (2)
C11_1	0.053 (3)	0.061 (4)	0.044 (3)	0.014 (3)	−0.006 (3)	−0.024 (3)
C12_1	0.048 (3)	0.070 (4)	0.030 (3)	0.007 (3)	−0.006 (2)	−0.014 (3)
C13_1	0.052 (3)	0.046 (3)	0.032 (3)	−0.001 (2)	−0.008 (2)	−0.005 (2)
C14_1	0.043 (3)	0.042 (3)	0.031 (2)	−0.001 (2)	−0.007 (2)	−0.010 (2)
Br1_2	0.0636 (4)	0.0754 (5)	0.0256 (3)	0.0077 (3)	−0.0095 (3)	−0.0158 (3)
Se1_2	0.0427 (3)	0.0443 (3)	0.0329 (3)	0.0030 (2)	−0.0072 (2)	−0.0131 (2)
N1_2	0.041 (2)	0.044 (2)	0.027 (2)	−0.0023 (18)	−0.0071 (17)	−0.0134 (17)

C1_2	0.039 (3)	0.049 (3)	0.030 (2)	−0.002 (2)	−0.007 (2)	−0.008 (2)
N2_2	0.0386 (19)	0.049 (2)	0.0271 (18)	−0.0029 (17)	−0.0078 (15)	−0.0119 (16)
C3_2	0.043 (3)	0.057 (4)	0.028 (2)	0.004 (3)	−0.008 (2)	−0.014 (2)
C3A_2	0.048 (3)	0.053 (3)	0.028 (2)	0.000 (2)	−0.010 (2)	−0.015 (2)
C4_2	0.051 (3)	0.064 (4)	0.032 (3)	0.004 (3)	−0.013 (2)	−0.019 (3)
C5_2	0.054 (3)	0.061 (3)	0.031 (3)	0.001 (3)	−0.012 (2)	−0.016 (2)
C6_2	0.054 (3)	0.061 (3)	0.028 (2)	−0.005 (3)	−0.009 (2)	−0.012 (2)
C7_2	0.047 (3)	0.059 (3)	0.029 (2)	−0.001 (3)	−0.008 (2)	−0.013 (2)
C7A_2	0.044 (3)	0.054 (3)	0.030 (2)	−0.001 (2)	−0.007 (2)	−0.014 (2)
O1_2	0.058 (3)	0.053 (2)	0.0291 (19)	−0.006 (2)	−0.0072 (18)	−0.0140 (18)
C1B_2	0.042 (5)	0.051 (5)	0.028 (5)	0.000 (5)	−0.009 (4)	−0.014 (5)
N2B_2	0.0386 (19)	0.049 (2)	0.0271 (18)	−0.0029 (17)	−0.0078 (15)	−0.0119 (16)
C3B_2	0.041 (5)	0.050 (5)	0.028 (5)	−0.003 (5)	−0.008 (5)	−0.011 (5)
C3AB_2	0.047 (4)	0.054 (4)	0.030 (4)	−0.001 (4)	−0.007 (4)	−0.012 (4)
C4B_2	0.048 (5)	0.057 (6)	0.032 (5)	−0.001 (5)	−0.008 (5)	−0.014 (5)
C5B_2	0.052 (5)	0.061 (5)	0.030 (5)	−0.001 (5)	−0.009 (5)	−0.016 (5)
C6B_2	0.052 (4)	0.059 (4)	0.029 (4)	−0.003 (4)	−0.009 (4)	−0.014 (4)
C7B_2	0.052 (4)	0.059 (4)	0.030 (4)	0.000 (4)	−0.010 (4)	−0.015 (4)
C7AB_2	0.048 (4)	0.056 (4)	0.029 (4)	0.001 (4)	−0.010 (4)	−0.014 (4)
O1B_2	0.050 (4)	0.055 (4)	0.029 (4)	−0.002 (4)	−0.007 (4)	−0.013 (4)
C8_2	0.042 (3)	0.032 (2)	0.028 (2)	−0.007 (2)	−0.006 (2)	−0.0052 (18)
C9_2	0.038 (3)	0.044 (3)	0.029 (2)	0.001 (2)	−0.008 (2)	−0.013 (2)
C10_2	0.051 (3)	0.041 (3)	0.035 (3)	−0.004 (2)	−0.012 (2)	−0.010 (2)
C11_2	0.054 (3)	0.041 (3)	0.033 (3)	0.001 (2)	−0.016 (2)	−0.014 (2)
C12_2	0.042 (3)	0.052 (3)	0.026 (2)	0.011 (2)	−0.011 (2)	−0.014 (2)
C13_2	0.046 (3)	0.055 (3)	0.032 (3)	−0.006 (3)	−0.004 (2)	−0.007 (2)
C14_2	0.042 (3)	0.048 (3)	0.034 (3)	−0.007 (2)	−0.008 (2)	−0.013 (2)

Geometric parameters (Å, º)

Br1_1—C12_1	1.908 (6)	Br1_2—C12_2	1.903 (5)
Se1_1—C8_1	1.862 (6)	Se1_2—C8_2	1.873 (5)
N1_1—C8_1	1.349 (7)	N1_2—C8_2	1.342 (7)
N1_1—C9_1	1.421 (6)	N1_2—C9_2	1.434 (6)
N1_1—H1_1	0.8800	N1_2—H1_2	0.8800
C1_1—N2_1	1.477 (10)	C1_2—N2_2	1.487 (9)
C1_1—C7A_1	1.528 (11)	C1_2—C7A_2	1.532 (10)
C1_1—H1A_1	0.9900	C1_2—H1A_2	0.9900
C1_1—H1B_1	0.9900	C1_2—H1B_2	0.9900
N2_1—C8_1	1.334 (7)	N2_2—C8_2	1.329 (6)
N2_1—C3_1	1.475 (11)	N2_2—C3_2	1.475 (8)
C3_1—C3A_1	1.505 (11)	C3_2—C3A_2	1.511 (9)
C3_1—H3A_1	0.9900	C3_2—H3A_2	0.9900
C3_1—H3B_1	0.9900	C3_2—H3B_2	0.9900
C3A_1—O1_1	1.455 (9)	C3A_2—O1_2	1.446 (9)
C3A_1—C4_1	1.503 (10)	C3A_2—C4_2	1.515 (9)
C3A_1—C7A_1	1.557 (10)	C3A_2—C7A_2	1.556 (9)
C4_1—C5_1	1.331 (11)	C4_2—C5_2	1.314 (10)
C4_1—H4_1	0.9500	C4_2—H4_2	0.9500
C5_1—C6_1	1.506 (12)	C5_2—C6_2	1.506 (10)
C5_1—H5_1	0.9500	C5_2—H5_2	0.9500
C6_1—O1_1	1.440 (9)	C6_2—O1_2	1.446 (8)

C6_1—C7_1	1.556 (12)	C6_2—C7_2	1.552 (10)
C6_1—H6_1	1.0000	C6_2—H6_2	1.0000
C7_1—C7A_1	1.540 (10)	C7_2—C7A_2	1.542 (9)
C7_1—H7A_1	0.9900	C7_2—H7A_2	0.9900
C7_1—H7B_1	0.9900	C7_2—H7B_2	0.9900
C7A_1—H7AA_1	1.0000	C7A_2—H7AA_2	1.0000
C1B_1—N2B_1	1.474 (18)	C1B_2—N2B_2	1.474 (18)
C1B_1—C7AB_1	1.521 (18)	C1B_2—C7AB_2	1.533 (19)
C1B_1—H1C_1	0.9900	C1B_2—H1C_2	0.9900
C1B_1—H1D_1	0.9900	C1B_2—H1D_2	0.9900
N2B_1—C8_1	1.334 (7)	N2B_2—C8_2	1.329 (6)
N2B_1—C3B_1	1.466 (17)	N2B_2—C3B_2	1.454 (18)
C3B_1—C3AB_1	1.504 (17)	C3B_2—C3AB_2	1.504 (18)
C3B_1—H3C_1	0.9900	C3B_2—H3C_2	0.9900
C3B_1—H3D_1	0.9900	C3B_2—H3D_2	0.9900
C3AB_1—O1B_1	1.441 (15)	C3AB_2—O1B_2	1.443 (17)
C3AB_1—C4B_1	1.497 (15)	C3AB_2—C4B_2	1.498 (16)
C3AB_1—C7AB_1	1.561 (15)	C3AB_2—C7AB_2	1.561 (16)
C4B_1—C5B_1	1.314 (16)	C4B_2—C5B_2	1.309 (17)
C4B_1—H4B_1	0.9500	C4B_2—H4B_2	0.9500
C5B_1—C6B_1	1.512 (17)	C5B_2—C6B_2	1.513 (18)
C5B_1—H5B_1	0.9500	C5B_2—H5B_2	0.9500
C6B_1—O1B_1	1.457 (15)	C6B_2—O1B_2	1.448 (16)
C6B_1—C7B_1	1.549 (17)	C6B_2—C7B_2	1.550 (19)
C6B_1—H6B_1	1.0000	C6B_2—H6B_2	1.0000
C7B_1—C7AB_1	1.555 (16)	C7B_2—C7AB_2	1.552 (17)
C7B_1—H7C_1	0.9900	C7B_2—H7C_2	0.9900
C7B_1—H7D_1	0.9900	C7B_2—H7D_2	0.9900
C7AB_1—H7AB_1	1.0000	C7AB_2—H7AB_2	1.0000
C9_1—C14_1	1.387 (8)	C9_2—C10_2	1.381 (8)
C9_1—C10_1	1.396 (8)	C9_2—C14_2	1.390 (8)
C10_1—C11_1	1.375 (8)	C10_2—C11_2	1.394 (8)
C10_1—H10_1	0.9500	C10_2—H10_2	0.9500
C11_1—C12_1	1.389 (10)	C11_2—C12_2	1.383 (9)
C11_1—H11_1	0.9500	C11_2—H11_2	0.9500
C12_1—C13_1	1.371 (9)	C12_2—C13_2	1.392 (9)
C13_1—C14_1	1.385 (7)	C13_2—C14_2	1.385 (8)
C13_1—H13_1	0.9500	C13_2—H13_2	0.9500
C14_1—H14_1	0.9500	C14_2—H14_2	0.9500
C8_1—N1_1—C9_1	126.4 (5)	C8_2—N1_2—C9_2	123.3 (4)
C8_1—N1_1—H1_1	116.8	C8_2—N1_2—H1_2	118.4
C9_1—N1_1—H1_1	116.8	C9_2—N1_2—H1_2	118.4
N2_1—C1_1—C7A_1	105.4 (7)	N2_2—C1_2—C7A_2	104.9 (6)
N2_1—C1_1—H1A_1	110.7	N2_2—C1_2—H1A_2	110.8
C7A_1—C1_1—H1A_1	110.7	C7A_2—C1_2—H1A_2	110.8
N2_1—C1_1—H1B_1	110.7	N2_2—C1_2—H1B_2	110.8
C7A_1—C1_1—H1B_1	110.7	C7A_2—C1_2—H1B_2	110.8
H1A_1—C1_1—H1B_1	108.8	H1A_2—C1_2—H1B_2	108.8
C8_1—N2_1—C3_1	123.8 (5)	C8_2—N2_2—C3_2	123.7 (5)
C8_1—N2_1—C1_1	124.7 (6)	C8_2—N2_2—C1_2	123.8 (5)
C3_1—N2_1—C1_1	111.4 (5)	C3_2—N2_2—C1_2	112.4 (5)

N2_1—C3_1—C3A_1	104.8 (7)	N2_2—C3_2—C3A_2	103.1 (6)
N2_1—C3_1—H3A_1	110.8	N2_2—C3_2—H3A_2	111.1
C3A_1—C3_1—H3A_1	110.8	C3A_2—C3_2—H3A_2	111.1
N2_1—C3_1—H3B_1	110.8	N2_2—C3_2—H3B_2	111.1
C3A_1—C3_1—H3B_1	110.8	C3A_2—C3_2—H3B_2	111.1
H3A_1—C3_1—H3B_1	108.9	H3A_2—C3_2—H3B_2	109.1
O1_1—C3A_1—C4_1	101.3 (6)	O1_2—C3A_2—C3_2	112.7 (6)
O1_1—C3A_1—C3_1	112.4 (7)	O1_2—C3A_2—C4_2	100.9 (5)
C4_1—C3A_1—C3_1	125.2 (7)	C3_2—C3A_2—C4_2	125.0 (6)
O1_1—C3A_1—C7A_1	99.3 (6)	O1_2—C3A_2—C7A_2	99.7 (5)
C4_1—C3A_1—C7A_1	109.4 (7)	C3_2—C3A_2—C7A_2	106.7 (5)
C3_1—C3A_1—C7A_1	106.2 (6)	C4_2—C3A_2—C7A_2	109.0 (6)
C5_1—C4_1—C3A_1	105.2 (7)	C5_2—C4_2—C3A_2	106.1 (6)
C5_1—C4_1—H4_1	127.4	C5_2—C4_2—H4_2	127.0
C3A_1—C4_1—H4_1	127.4	C3A_2—C4_2—H4_2	127.0
C4_1—C5_1—C6_1	106.1 (7)	C4_2—C5_2—C6_2	105.5 (6)
C4_1—C5_1—H5_1	126.9	C4_2—C5_2—H5_2	127.2
C6_1—C5_1—H5_1	126.9	C6_2—C5_2—H5_2	127.2
O1_1—C6_1—C5_1	101.7 (7)	O1_2—C6_2—C5_2	101.3 (6)
O1_1—C6_1—C7_1	100.6 (6)	O1_2—C6_2—C7_2	101.0 (5)
C5_1—C6_1—C7_1	108.0 (7)	C5_2—C6_2—C7_2	109.0 (7)
O1_1—C6_1—H6_1	115.0	O1_2—C6_2—H6_2	114.6
C5_1—C6_1—H6_1	115.0	C5_2—C6_2—H6_2	114.6
C7_1—C6_1—H6_1	115.0	C7_2—C6_2—H6_2	114.6
C7A_1—C7_1—C6_1	100.5 (6)	C7A_2—C7_2—C6_2	100.5 (6)
C7A_1—C7_1—H7A_1	111.7	C7A_2—C7_2—H7A_2	111.7
C6_1—C7_1—H7A_1	111.7	C6_2—C7_2—H7A_2	111.7
C7A_1—C7_1—H7B_1	111.7	C7A_2—C7_2—H7B_2	111.7
C6_1—C7_1—H7B_1	111.7	C6_2—C7_2—H7B_2	111.7
H7A_1—C7_1—H7B_1	109.4	H7A_2—C7_2—H7B_2	109.4
C1_1—C7A_1—C7_1	117.9 (7)	C1_2—C7A_2—C7_2	117.3 (7)
C1_1—C7A_1—C3A_1	102.5 (6)	C1_2—C7A_2—C3A_2	101.8 (6)
C7_1—C7A_1—C3A_1	101.8 (6)	C7_2—C7A_2—C3A_2	101.6 (5)
C1_1—C7A_1—H7AA_1	111.3	C1_2—C7A_2—H7AA_2	111.7
C7_1—C7A_1—H7AA_1	111.3	C7_2—C7A_2—H7AA_2	111.7
C3A_1—C7A_1—H7AA_1	111.3	C3A_2—C7A_2—H7AA_2	111.7
C6_1—O1_1—C3A_1	95.5 (5)	C6_2—O1_2—C3A_2	95.2 (5)
N2B_1—C1B_1—C7AB_1	101.4 (13)	N2B_2—C1B_2—C7AB_2	99.9 (14)
N2B_1—C1B_1—H1C_1	111.5	N2B_2—C1B_2—H1C_2	111.8
C7AB_1—C1B_1—H1C_1	111.5	C7AB_2—C1B_2—H1C_2	111.8
N2B_1—C1B_1—H1D_1	111.5	N2B_2—C1B_2—H1D_2	111.8
C7AB_1—C1B_1—H1D_1	111.5	C7AB_2—C1B_2—H1D_2	111.8
H1C_1—C1B_1—H1D_1	109.3	H1C_2—C1B_2—H1D_2	109.5
C8_1—N2B_1—C3B_1	119.5 (8)	C8_2—N2B_2—C3B_2	122.3 (10)
C8_1—N2B_1—C1B_1	125.0 (9)	C8_2—N2B_2—C1B_2	119.6 (10)
C3B_1—N2B_1—C1B_1	115.5 (10)	C3B_2—N2B_2—C1B_2	117.3 (12)
N2B_1—C3B_1—C3AB_1	101.6 (12)	N2B_2—C3B_2—C3AB_2	99.2 (13)
N2B_1—C3B_1—H3C_1	111.4	N2B_2—C3B_2—H3C_2	111.9
C3AB_1—C3B_1—H3C_1	111.4	C3AB_2—C3B_2—H3C_2	111.9
N2B_1—C3B_1—H3D_1	111.4	N2B_2—C3B_2—H3D_2	111.9
C3AB_1—C3B_1—H3D_1	111.4	C3AB_2—C3B_2—H3D_2	111.9
H3C_1—C3B_1—H3D_1	109.3	H3C_2—C3B_2—H3D_2	109.6

O1B_1—C3AB_1—C4B_1	102.2 (12)	O1B_2—C3AB_2—C4B_2	102.1 (14)
O1B_1—C3AB_1—C3B_1	114.2 (16)	O1B_2—C3AB_2—C3B_2	113 (2)
C4B_1—C3AB_1—C3B_1	123.5 (14)	C4B_2—C3AB_2—C3B_2	121.6 (18)
O1B_1—C3AB_1—C7AB_1	99.5 (12)	O1B_2—C3AB_2—C7AB_2	98.6 (14)
C4B_1—C3AB_1—C7AB_1	108.3 (13)	C4B_2—C3AB_2—C7AB_2	109.8 (17)
C3B_1—C3AB_1—C7AB_1	106.6 (12)	C3B_2—C3AB_2—C7AB_2	109.4 (15)
C5B_1—C4B_1—C3AB_1	105.9 (13)	C5B_2—C4B_2—C3AB_2	104.4 (14)
C5B_1—C4B_1—H4B_1	127.0	C5B_2—C4B_2—H4B_2	127.8
C3AB_1—C4B_1—H4B_1	127.0	C3AB_2—C4B_2—H4B_2	127.8
C4B_1—C5B_1—C6B_1	106.1 (13)	C4B_2—C5B_2—C6B_2	107.1 (15)
C4B_1—C5B_1—H5B_1	126.9	C4B_2—C5B_2—H5B_2	126.5
C6B_1—C5B_1—H5B_1	126.9	C6B_2—C5B_2—H5B_2	126.5
O1B_1—C6B_1—C5B_1	100.9 (13)	O1B_2—C6B_2—C5B_2	102.7 (15)
O1B_1—C6B_1—C7B_1	100.8 (13)	O1B_2—C6B_2—C7B_2	99.0 (15)
C5B_1—C6B_1—C7B_1	107.6 (16)	C5B_2—C6B_2—C7B_2	107.4 (18)
O1B_1—C6B_1—H6B_1	115.2	O1B_2—C6B_2—H6B_2	115.3
C5B_1—C6B_1—H6B_1	115.2	C5B_2—C6B_2—H6B_2	115.3
C7B_1—C6B_1—H6B_1	115.2	C7B_2—C6B_2—H6B_2	115.3
C6B_1—C7B_1—C7AB_1	100.6 (12)	C6B_2—C7B_2—C7AB_2	99.5 (13)
C6B_1—C7B_1—H7C_1	111.7	C6B_2—C7B_2—H7C_2	111.9
C7AB_1—C7B_1—H7C_1	111.7	C7AB_2—C7B_2—H7C_2	111.9
C6B_1—C7B_1—H7D_1	111.7	C6B_2—C7B_2—H7D_2	111.9
C7AB_1—C7B_1—H7D_1	111.7	C7AB_2—C7B_2—H7D_2	111.9
H7C_1—C7B_1—H7D_1	109.4	H7C_2—C7B_2—H7D_2	109.6
C1B_1—C7AB_1—C7B_1	118.0 (17)	C1B_2—C7AB_2—C7B_2	115 (2)
C1B_1—C7AB_1—C3AB_1	102.8 (13)	C1B_2—C7AB_2—C3AB_2	100.8 (15)
C7B_1—C7AB_1—C3AB_1	101.3 (11)	C7B_2—C7AB_2—C3AB_2	102.1 (13)
C1B_1—C7AB_1—H7AB_1	111.3	C1B_2—C7AB_2—H7AB_2	112.7
C7B_1—C7AB_1—H7AB_1	111.3	C7B_2—C7AB_2—H7AB_2	112.7
C3AB_1—C7AB_1—H7AB_1	111.3	C3AB_2—C7AB_2—H7AB_2	112.7
C3AB_1—O1B_1—C6B_1	95.5 (11)	C3AB_2—O1B_2—C6B_2	95.4 (12)
N2B_1—C8_1—N1_1	117.0 (5)	N2B_2—C8_2—N1_2	118.0 (5)
N2_1—C8_1—N1_1	117.0 (5)	N2_2—C8_2—N1_2	118.0 (5)
N2B_1—C8_1—Se1_1	121.1 (4)	N2B_2—C8_2—Se1_2	121.4 (4)
N2_1—C8_1—Se1_1	121.1 (4)	N2_2—C8_2—Se1_2	121.4 (4)
N1_1—C8_1—Se1_1	121.9 (4)	N1_2—C8_2—Se1_2	120.5 (4)
C14_1—C9_1—C10_1	119.8 (5)	C10_2—C9_2—C14_2	121.3 (5)
C14_1—C9_1—N1_1	118.6 (5)	C10_2—C9_2—N1_2	119.3 (5)
C10_1—C9_1—N1_1	121.5 (5)	C14_2—C9_2—N1_2	119.4 (5)
C11_1—C10_1—C9_1	120.0 (5)	C9_2—C10_2—C11_2	119.3 (5)
C11_1—C10_1—H10_1	120.0	C9_2—C10_2—H10_2	120.3
C9_1—C10_1—H10_1	120.0	C11_2—C10_2—H10_2	120.3
C10_1—C11_1—C12_1	119.1 (6)	C12_2—C11_2—C10_2	119.0 (5)
C10_1—C11_1—H11_1	120.4	C12_2—C11_2—H11_2	120.5
C12_1—C11_1—H11_1	120.4	C10_2—C11_2—H11_2	120.5
C13_1—C12_1—C11_1	121.8 (5)	C11_2—C12_2—C13_2	122.0 (5)
C13_1—C12_1—Br1_1	118.8 (5)	C11_2—C12_2—Br1_2	118.5 (4)
C11_1—C12_1—Br1_1	119.4 (5)	C13_2—C12_2—Br1_2	119.5 (4)
C12_1—C13_1—C14_1	118.9 (5)	C14_2—C13_2—C12_2	118.6 (5)
C12_1—C13_1—H13_1	120.5	C14_2—C13_2—H13_2	120.7
C14_1—C13_1—H13_1	120.5	C12_2—C13_2—H13_2	120.7
C13_1—C14_1—C9_1	120.4 (5)	C13_2—C14_2—C9_2	119.8 (5)

C13_1—C14_1—H14_1	119.8	C13_2—C14_2—H14_2	120.1
C9_1—C14_1—H14_1	119.8	C9_2—C14_2—H14_2	120.1

C7A_1—C1_1—N2_1—C8_1	163.1 (6)	C7A_2—C1_2—N2_2—C8_2	163.4 (6)
C7A_1—C1_1—N2_1—C3_1	−14.2 (9)	C7A_2—C1_2—N2_2—C3_2	−11.5 (11)
C8_1—N2_1—C3_1—C3A_1	177.0 (6)	C8_2—N2_2—C3_2—C3A_2	175.4 (5)
C1_1—N2_1—C3_1—C3A_1	−5.7 (10)	C1_2—N2_2—C3_2—C3A_2	−9.8 (9)
N2_1—C3_1—C3A_1—O1_1	−84.5 (9)	N2_2—C3_2—C3A_2—O1_2	−81.3 (7)
N2_1—C3_1—C3A_1—C4_1	152.1 (8)	N2_2—C3_2—C3A_2—C4_2	155.8 (7)
N2_1—C3_1—C3A_1—C7A_1	23.0 (10)	N2_2—C3_2—C3A_2—C7A_2	27.1 (8)
O1_1—C3A_1—C4_1—C5_1	34.3 (9)	O1_2—C3A_2—C4_2—C5_2	32.5 (8)
C3_1—C3A_1—C4_1—C5_1	162.4 (9)	C3_2—C3A_2—C4_2—C5_2	160.4 (7)
C7A_1—C3A_1—C4_1—C5_1	−69.9 (9)	C7A_2—C3A_2—C4_2—C5_2	−71.8 (8)
C3A_1—C4_1—C5_1—C6_1	−2.2 (10)	C3A_2—C4_2—C5_2—C6_2	0.7 (9)
C4_1—C5_1—C6_1—O1_1	−31.0 (9)	C4_2—C5_2—C6_2—O1_2	−33.7 (8)
C4_1—C5_1—C6_1—C7_1	74.3 (9)	C4_2—C5_2—C6_2—C7_2	72.3 (8)
O1_1—C6_1—C7_1—C7A_1	36.7 (8)	O1_2—C6_2—C7_2—C7A_2	35.7 (7)
C5_1—C6_1—C7_1—C7A_1	−69.4 (8)	C5_2—C6_2—C7_2—C7A_2	−70.5 (7)
N2_1—C1_1—C7A_1—C7_1	137.9 (7)	N2_2—C1_2—C7A_2—C7_2	136.7 (7)
N2_1—C1_1—C7A_1—C3A_1	27.2 (8)	N2_2—C1_2—C7A_2—C3A_2	26.9 (10)
C6_1—C7_1—C7A_1—C1_1	−110.7 (8)	C6_2—C7_2—C7A_2—C1_2	−108.5 (8)
C6_1—C7_1—C7A_1—C3A_1	0.5 (8)	C6_2—C7_2—C7A_2—C3A_2	1.4 (7)
O1_1—C3A_1—C7A_1—C1_1	85.4 (7)	O1_2—C3A_2—C7A_2—C1_2	83.4 (7)
C4_1—C3A_1—C7A_1—C1_1	−169.0 (7)	C3_2—C3A_2—C7A_2—C1_2	−34.0 (9)
C3_1—C3A_1—C7A_1—C1_1	−31.2 (9)	C4_2—C3A_2—C7A_2—C1_2	−171.4 (7)
O1_1—C3A_1—C7A_1—C7_1	−36.9 (7)	O1_2—C3A_2—C7A_2—C7_2	−37.9 (6)
C4_1—C3A_1—C7A_1—C7_1	68.7 (8)	C3_2—C3A_2—C7A_2—C7_2	−155.3 (6)
C3_1—C3A_1—C7A_1—C7_1	−153.6 (8)	C4_2—C3A_2—C7A_2—C7_2	67.2 (7)
C5_1—C6_1—O1_1—C3A_1	49.9 (7)	C5_2—C6_2—O1_2—C3A_2	51.6 (6)
C7_1—C6_1—O1_1—C3A_1	−61.2 (7)	C7_2—C6_2—O1_2—C3A_2	−60.6 (6)
C4_1—C3A_1—O1_1—C6_1	−51.4 (7)	C3_2—C3A_2—O1_2—C6_2	173.7 (6)
C3_1—C3A_1—O1_1—C6_1	172.7 (7)	C4_2—C3A_2—O1_2—C6_2	−50.8 (6)
C7A_1—C3A_1—O1_1—C6_1	60.7 (7)	C7A_2—C3A_2—O1_2—C6_2	60.8 (6)
C7AB_1—C1B_1—N2B_1—C8_1	−158.9 (12)	C7AB_2—C1B_2—N2B_2—C8_2	−162.8 (15)
C7AB_1—C1B_1—N2B_1—C3B_1	20 (3)	C7AB_2—C1B_2—N2B_2—C3B_2	27 (4)
C8_1—N2B_1—C3B_1—C3AB_1	−179.5 (11)	C8_2—N2B_2—C3B_2—C3AB_2	−176.0 (14)
C1B_1—N2B_1—C3B_1—C3AB_1	2 (3)	C1B_2—N2B_2—C3B_2—C3AB_2	−6 (4)
N2B_1—C3B_1—C3AB_1—O1B_1	86.4 (19)	N2B_2—C3B_2—C3AB_2—O1B_2	91 (3)
N2B_1—C3B_1—C3AB_1—C4B_1	−148.5 (17)	N2B_2—C3B_2—C3AB_2—C4B_2	−147 (3)
N2B_1—C3B_1—C3AB_1—C7AB_1	−22 (2)	N2B_2—C3B_2—C3AB_2—C7AB_2	−18 (4)
O1B_1—C3AB_1—C4B_1—C5B_1	−32.3 (19)	O1B_2—C3AB_2—C4B_2—C5B_2	−39 (3)
C3B_1—C3AB_1—C4B_1—C5B_1	−162 (2)	C3B_2—C3AB_2—C4B_2—C5B_2	−166 (3)
C7AB_1—C3AB_1—C4B_1—C5B_1	72.2 (19)	C7AB_2—C3AB_2—C4B_2—C5B_2	65 (3)
C3AB_1—C4B_1—C5B_1—C6B_1	0 (2)	C3AB_2—C4B_2—C5B_2—C6B_2	9 (3)
C4B_1—C5B_1—C6B_1—O1B_1	32.4 (19)	C4B_2—C5B_2—C6B_2—O1B_2	24 (3)
C4B_1—C5B_1—C6B_1—C7B_1	−72.8 (19)	C4B_2—C5B_2—C6B_2—C7B_2	−80 (3)
O1B_1—C6B_1—C7B_1—C7AB_1	−35.3 (19)	O1B_2—C6B_2—C7B_2—C7AB_2	−41 (2)
C5B_1—C6B_1—C7B_1—C7AB_1	70.0 (18)	C5B_2—C6B_2—C7B_2—C7AB_2	66 (2)
N2B_1—C1B_1—C7AB_1—C7B_1	−141.7 (18)	N2B_2—C1B_2—C7AB_2—C7B_2	−143 (2)
N2B_1—C1B_1—C7AB_1—C3AB_1	−31 (2)	N2B_2—C1B_2—C7AB_2—C3AB_2	−34 (3)
C6B_1—C7B_1—C7AB_1—C1B_1	110 (2)	C6B_2—C7B_2—C7AB_2—C1B_2	112 (3)
C6B_1—C7B_1—C7AB_1—C3AB_1	−1.6 (19)	C6B_2—C7B_2—C7AB_2—C3AB_2	4 (2)

O1B_1—C3AB_1—C7AB_1—C1B_1	−84.0 (18)	O1B_2—C3AB_2—C7AB_2—C1B_2	−84 (2)
C4B_1—C3AB_1—C7AB_1—C1B_1	169.6 (18)	C4B_2—C3AB_2—C7AB_2—C1B_2	170 (2)
C3B_1—C3AB_1—C7AB_1—C1B_1	35 (2)	C3B_2—C3AB_2—C7AB_2—C1B_2	34 (3)
O1B_1—C3AB_1—C7AB_1—C7B_1	38.3 (16)	O1B_2—C3AB_2—C7AB_2—C7B_2	34 (2)
C4B_1—C3AB_1—C7AB_1—C7B_1	−68.0 (17)	C4B_2—C3AB_2—C7AB_2—C7B_2	−72 (2)
C3B_1—C3AB_1—C7AB_1—C7B_1	157.2 (18)	C3B_2—C3AB_2—C7AB_2—C7B_2	152 (3)
C4B_1—C3AB_1—O1B_1—C6B_1	50.0 (15)	C4B_2—C3AB_2—O1B_2—C6B_2	51.3 (18)
C3B_1—C3AB_1—O1B_1—C6B_1	−174.3 (13)	C3B_2—C3AB_2—O1B_2—C6B_2	−176.6 (16)
C7AB_1—C3AB_1—O1B_1—C6B_1	−61.2 (13)	C7AB_2—C3AB_2—O1B_2—C6B_2	−61.2 (16)
C5B_1—C6B_1—O1B_1—C3AB_1	−49.7 (14)	C5B_2—C6B_2—O1B_2—C3AB_2	−45.4 (19)
C7B_1—C6B_1—O1B_1—C3AB_1	60.8 (16)	C7B_2—C6B_2—O1B_2—C3AB_2	64.8 (17)
C3B_1—N2B_1—C8_1—N1_1	174.7 (15)	C3B_2—N2B_2—C8_2—N1_2	−1 (3)
C1B_1—N2B_1—C8_1—N1_1	−7 (2)	C1B_2—N2B_2—C8_2—N1_2	−170 (2)
C3B_1—N2B_1—C8_1—Se1_1	−2.7 (16)	C3B_2—N2B_2—C8_2—Se1_2	177 (3)
C1B_1—N2B_1—C8_1—Se1_1	175.8 (19)	C1B_2—N2B_2—C8_2—Se1_2	8 (2)
C3_1—N2_1—C8_1—N1_1	3.4 (9)	C3_2—N2_2—C8_2—N1_2	174.4 (6)
C1_1—N2_1—C8_1—N1_1	−173.5 (6)	C1_2—N2_2—C8_2—N1_2	0.1 (10)
C3_1—N2_1—C8_1—Se1_1	−174.0 (7)	C3_2—N2_2—C8_2—Se1_2	−7.5 (8)
C1_1—N2_1—C8_1—Se1_1	9.1 (8)	C1_2—N2_2—C8_2—Se1_2	178.2 (7)
C9_1—N1_1—C8_1—N2B_1	171.6 (5)	C9_2—N1_2—C8_2—N2B_2	−175.2 (5)
C9_1—N1_1—C8_1—N2_1	171.6 (5)	C9_2—N1_2—C8_2—N2_2	−175.2 (5)
C9_1—N1_1—C8_1—Se1_1	−11.0 (7)	C9_2—N1_2—C8_2—Se1_2	6.7 (7)
C8_1—N1_1—C9_1—C14_1	−115.1 (6)	C8_2—N1_2—C9_2—C10_2	−98.1 (6)
C8_1—N1_1—C9_1—C10_1	67.9 (8)	C8_2—N1_2—C9_2—C14_2	83.8 (7)
C14_1—C9_1—C10_1—C11_1	−2.0 (9)	C14_2—C9_2—C10_2—C11_2	−1.7 (8)
N1_1—C9_1—C10_1—C11_1	174.9 (6)	N1_2—C9_2—C10_2—C11_2	−179.8 (5)
C9_1—C10_1—C11_1—C12_1	0.5 (10)	C9_2—C10_2—C11_2—C12_2	1.1 (8)
C10_1—C11_1—C12_1—C13_1	0.6 (11)	C10_2—C11_2—C12_2—C13_2	−0.2 (8)
C10_1—C11_1—C12_1—Br1_1	−177.9 (5)	C10_2—C11_2—C12_2—Br1_2	−179.4 (4)
C11_1—C12_1—C13_1—C14_1	−0.2 (10)	C11_2—C12_2—C13_2—C14_2	−0.1 (9)
Br1_1—C12_1—C13_1—C14_1	178.4 (5)	Br1_2—C12_2—C13_2—C14_2	179.0 (4)
C12_1—C13_1—C14_1—C9_1	−1.4 (9)	C12_2—C13_2—C14_2—C9_2	−0.4 (9)
C10_1—C9_1—C14_1—C13_1	2.5 (9)	C10_2—C9_2—C14_2—C13_2	1.4 (9)
N1_1—C9_1—C14_1—C13_1	−174.5 (5)	N1_2—C9_2—C14_2—C13_2	179.4 (5)

Hydrogen-bond geometry (Å, º)

Cg8 and Cg17 are the centroids of the C9_1···C14_1 and C9_2···C14_2 rings.

D—H···*A*	*D*—H	H···*A*	*D*···*A*	*D*—H···*A*
N1_1—H1_1···Se1_2^{i}	0.88	2.67	3.460 (5)	151
C11_1—H11_1···O1_1^{i}	0.95	2.34	3.283 (8)	174
C11_1—H11_1···O1*B*_1^{i}	0.95	2.25	3.176 (14)	165
N1_2—H1_2···Se1_1^{ii}	0.88	2.64	3.393 (5)	144
C11_2—H11_2···O1_2^{iii}	0.95	2.44	3.368 (7)	167
C11_2—H11_2···O1*B*_2^{iii}	0.95	2.48	3.36 (2)	154
C1_1—H1*A*_1···*Cg*17^{ii}	0.99	2.83	3.720 (11)	150
C3_2—H3*B*_2···*Cg*8^{i}	0.99	2.81	3.724 (9)	154

Symmetry codes: (i) −*x*, −*y*+1, −*z*+1; (ii) −*x*+1, −*y*+1, −*z*+1; (iii) −*x*+1, −*y*+2, −*z*+1.
